# Advances in GPCR-Targeted PET Radiotracer Patents (2020–2025)

**DOI:** 10.3390/ph19060900

**Published:** 2026-06-05

**Authors:** Rebecca Ferrisi, Clara Mocchetti, Alessia Cazzaniga, Marco De Amici, Claudio Papotto, Clelia Dallanoce

**Affiliations:** 1 Department of Pharmaceutical Sciences, Medicinal Chemistry Section “Pietro Pratesi”, University of Milan, Via L. Mangiagalli 25, 20133 Milan, Italy; clara.mocchetti@unimi.it (C.M.); alessia.cazzaniga@unimi.it (A.C.); marco.deamici@unimi.it (M.D.A.); clelia.dallanoce@unimi.it (C.D.); 2 Tecnomed Foundation, University of Milan-Bicocca, Via G. Pergolesi 33, 20900 Monza, Italy; claudio.papotto@unimib.it

**Keywords:** positron emission tomography, G protein-coupled receptors, radioligands, GPCR-targeted tracers, molecular imaging, tracer development

## Abstract

**Background:** Positron emission tomography (PET) is a molecular imaging technique that exploits the β^+^ decay of selected radionuclides to enable non-invasive in vivo investigation of biochemical and physiological processes, including early and subclinical disease alterations. Radiotracers are designed to bind specific molecular targets with high affinity and selectivity. Among the targets to which PET devotes increasing attention are G protein-coupled receptors (GPCRs)—the largest class of transmembrane receptors—which orchestrate a wide spectrum of biological outcomes and are widely implicated in human disease. **Objectives:** This review analyzes patents published between 2020 and 2025 focusing on GPCR-targeted PET radiotracers, highlighting design strategies, radionuclide selection, and translational perspectives across oncology, central nervous system (CNS) disorders, and inflammatory diseases. **Results:** Patent activity shows that most GPCR-targeted PET tracers are derived from validated ligands adapted for imaging while preserving affinity and selectivity. Oncology patents mainly favor peptide-based or modular metal–chelator platforms enabling radionuclide flexibility and theranostic extension, whereas CNS tracers rely on drug-like small molecules optimized under strict ADME and blood–brain barrier constraints. Increasing emphasis on non-orthosteric, function-sensitive, and dual-targeting approaches reflects a shift toward interrogating GPCR signaling states, while inflammatory indications remain comparatively underrepresented despite clear biological foundations. **Conclusions:** Current patent trends consolidate GPCR-targeted PET tracers as well-established diagnostic tools while progressively expanding their clinical utility, both as platforms supporting translational research—informing mechanistic insight and drug development—and as components of emerging theranostic strategies across multiple disease areas.

## 1. Introduction

**Figure 1:**
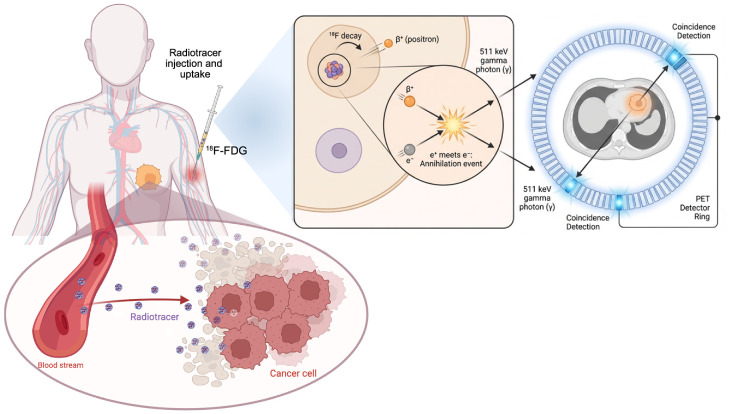
Schematic representation of the physical principles underlying positron emission tomography (PET). (i) Radiotracer injection and uptake: As an example, ^18^F-FDG is injected and accumulates in metabolically active tissues such as tumors. (ii) Positron emission: The ^18^F nucleus incorporated in the radiotracer undergoes β^+^ decay, emitting a positron (β^+^). The latter travels a short distance through the surrounding tissue while losing kinetic energy. (iii) Annihilation event: The positron (e^+^) encounters an electron (e^−^), resulting in mutual annihilation and conversion of their mass into two 511-keV γ photons emitted ~180° apart. (iv) Coincidence detection and image reconstruction: These coincident photons are detected by opposing scintillation detectors in the PET scanner, enabling reconstruction of the line of response and, through computational algorithms, the three-dimensional distribution of the radiotracer in vivo. Part of this figure was created with Biorender.com.

Positron emission tomography (PET) is an advanced imaging technique in nuclear medicine that enables the study of physiological and pathophysiological processes in various organs of the body. It involves the administration of a radiopharmaceutical—a chemical entity, just like other medicinal molecules or xenobiotics in general, containing one radioactive atom—that accumulates in specific areas of the body based on its biochemical properties. The isotope within the radiopharmaceutical undergoes β^+^ decay, emitting a positron that subsequently interacts with a nearby electron in the surrounding tissue. This interaction results in an annihilation event, producing two 511-keV photons emitted in opposite trajectories, which are detected by a scintillator in the scanning device (Figure 1). Sophisticated computational algorithms then reconstruct the photon trajectories to create a three-dimensional image, providing a quantitative map of radiotracer uptake in specific tissues. This enables the in vivo assessment of biochemical and physiological processes, which, in cases of aberrations, may occur prior to the appearance of macroscopic anatomical signs of a disease [1,2].

A variety of radionuclides is used in nuclear medicine. This broad family can be divided into mainly therapeutic and diagnostic nuclides. Therapeutic radionuclides are primarily particle emitters (α, β^−^ and, in some cases, Auger electrons) used in targeted radionuclide therapy (e.g., ^225^Ac, ^32^P, ^177^Lu, ^131^I). Diagnostic nuclides include γ-emitters (for scintigraphy and SPECT) and positron-emitters (PET). Notably, some radionuclides (e.g., ^177^Lu and ^131^I) show both therapeutic particle emissions and useful γ emissions, enabling post-therapy imaging/dosimetry.

In the context of PET, fluorine-18 (^18^F) and carbon-11 (^11^C) are among the most widely used β^+^-emitting radionuclides (Table 1). ^18^F offers several advantages over other PET radionuclides, including a short positron range (<1 mm), which enables high-resolution image acquisition, a favorable decay profile (97% positron emission and 3% electron capture), and an optimal physical half-life of 109.8 min, allowing distribution to satellite facilities without an on-site cyclotron [3]. Although ^18^F is highly competitive and often preferred for peptides and small biomolecules, it is not the first choice radionuclide for larger biomolecules such as antibodies, nanobodies, proteins, or oligonucleotides, where longer-lived radionuclides like ^89^Zr (78 h) and ^64^Cu (12.7 h) are more suitable to match their slower pharmacokinetics and enable imaging at 24–72 h post-injection [4]. ^18^F is by far the most widely used radionuclide in oncology, primarily owing to 2-deoxy-2-[^18^F]fluoroglucose or [^18^F]Fluorodeoxyglucose ([^18^F]FDG), a glucose analogue bearing a bioisosteric substitution of the hydroxyl group at the 2-position with ^18^F, which represents the universal standard for oncologic PET imaging [5]. ^11^C presents its own strengths, enabling the labeling of drugs and ligands in native form without altering their pharmacological properties; its short half-life (~20 min) allows multiple same-day scans with low radiation burden, and advances in radiochemistry now permit incorporation into diverse functional groups—though its use remains limited by the need for on-site cyclotron production and rapid synthesis. ^11^C-radioligands are particularly impactful in neurology and cardiology, where they provide unique tools for studying neurotransmitter systems, protein aggregates, and brain metabolism, as well as myocardial metabolism and innervation [6]. Other positron-emitters are commonly used for their peculiar characteristics in PET imaging, as listed in Table 1. Among them, nitrogen-13 (^13^N, half-life t_1/2_ = 10 min) and oxygen-15 (^15^O, half-life t_1/2_ = 2 min) are short-lived PET radionuclides, thus making them unsuitable for multiple-step radiosynthesis in most scenarios. However, they find clinical application in perfusion and metabolic studies, where rapid tracer kinetics are advantageous.

In addition to organic radionuclides, radiometals broaden the scope of PET by enabling the labeling of complex biological vectors, including peptides, proteins, and antibodies. Notable examples are gallium-68 (^68^Ga, t_1_/_2_ = 67.7 min), copper-64 (^64^Cu, t_1_/_2_ = 12.7 h), and zirconium-89 (^89^Zr, t_1_/_2_ = 78.4 h). Their incorporation relies on coordination chemistry, in which the radiometal is complexed with a suitable chelator that is subsequently conjugated to the targeting vector. The generally mild labeling conditions and the broad range of available half-lives—from a few hours to several days—allow selection of the most appropriate radionuclide according to the intended clinical or research application [7]. Notably, radiometals and longer-lived radionuclides provide an ideal framework for theranostic applications, relying on matched pairs of agents to enable both diagnosis and treatment of tumors on the same biological and molecular basis. To do so, radionuclides with different properties are attached to similar—or ideally identical—scaffolds, which can bind the target of interest with high affinity and thereby exert either diagnostic or therapeutic activity. Unlike diagnostic isotopes, which primarily decay through β^+^ or γ-emission, therapeutic radionuclides act by releasing cytotoxic radiation characterized either by a very high energy but an extremely short tissue range (α particles, Auger electrons), or by moderately energetic β^−^ emissions with longer penetration in tissues. Such radiation is theoretically capable of directly damaging the DNA of cancer cells, thereby impairing their ability to replicate and inducing apoptosis.

The cornerstone of the modern theranostics is lutetium-177 (^177^Lu), a therapeutic β^−^-emitting radionuclide with a tissue penetration of 1–2 mm, making it suitable for targeting small tumors or micrometastases while limiting damage to surrounding tissues. In addition, it emits low energy γ radiation, which is useful for imaging and dosimetry during therapy. Clinically, ^177^Lu has been successfully employed in the development of radiopharmaceuticals such as [^177^Lu]Lu-DOTATATE (Lutathera^®^) for neuroendocrine tumors (NETs) and [^177^Lu]Lu-PSMA-617 (Pluvicto^®^) for prostate cancer, which have set new standards in targeted radionuclide therapy. Both agents can form theranostic pairs: the vector (DOTATATE) can be labeled with a PET radionuclide (^68^Ga or ^64^Cu) to identify patients with somatostatin receptor–positive tumors prior to therapy with Lutathera^®^. Similarly, PET imaging with [^68^Ga]Ga-PSMA-11 (or [^18^F]-labeled PSMA ligands) allows the selection of prostate cancer patients eligible for treatment with Pluvicto^®^. Other emerging theranostic combinations are ^124^I/^131^I for thyroid cancer and ^86^Y/^90^Y for dosimetry and therapy [8].

**Table 1:** Overview of commonly employed positron-emitting isotopes.

Isotope	Half-Life	Mode of Decay	Main Application Area	Clinical Examples of PET Pharmaceuticals
^18^F	109.8 min	β+ (97%), EC (3%)	Oncology Neurology	▪ [^18^F]FDG—reference tracer; gold standard in oncology (solid tumors, lymphomas); also used in infection/inflammation [9]. ▪ [^18^F]FLT—investigational tracer for cellular proliferation and early therapy response [10]. ▪ [^18^F]FET—widely used for brain tumors (gliomas) [11]. ▪ [^18^F]NaF—bone imaging [12], and vascular disorders [13]. ▪ [^18^F]PSMA and [^18^F]fluciclovine—prostate cancer [14]. ▪ [^18^F]fluoroestradiol—estrogen receptor (ER)-positive breast cancer [15]. ▪ [^18^F]FDOPA—dopaminergic nerve terminals in the striatum of patients with suspected Parkinsonian syndrome [16]. ▪ [^18^F]flortaucipir –aggregated tau neurofibrillary tangles in Alzheimer’s disease [17].
^11^C	20.4 min	β+ (100%)	Neurology Cardiology	▪ [^11^C]raclopride—striatal dopamine binding in autism spectrum disorder [18]. ▪ [^11^C]PIB—β-amyloid deposits in Alzheimer’s disease [19]. ▪ [^11^C]acetate—measures oxidative metabolism and myocardial perfusion; also applied in oncology (e.g., bladder carcinoma, brain tumors) [20].
^13^N	10 min	β+ (100%)	Cardiology	▪ [^13^N]NH_3_—myocardial perfusion imaging (rest/stress) for ischemic heart disease [21].
^15^O	2 min	β+ (100%)	Cardiology & Neurology (perfusion studies)	▪ [^15^O]H_2_O—gold standard tracer for quantitative myocardial and cerebral perfusion imaging [22].
^124^I	4.2 d	β+ (23%), EC (77%)	Oncology	▪ [^124^I]NaI—PET surrogate for ^131^I therapy, used for thyroid imaging, diagnosis, and dosimetry in differentiated thyroid cancer and hyperthyroidism [23,24].
^44^Sc	4.0 h	β+ (94%), EC (6%)	Oncology	▪ [^44^Sc]ScDOTATOC/-TATE—NETs [25]. ▪ [^44^Sc]ScPSMA-617—prostate carcinoma [26].
^64^Cu	12.7 h	β+ (17%), EC (44%), β− (39%)	Oncology	▪ [^64^Cu]CuDOTATATE—NETs [27]. ▪ [^64^Cu]CuPSMA-617– prostate carcinoma [28].
^68^Ga	67.7 min	β+ (89%), EC (11%)	Oncology	▪ [^68^Ga]GaDOTA-TATE/-TOC/-NOC—NETs [29]. ▪ [^68^Ga]GaPSMA-11—prostate cancer [30].
^82^Rb	1.3 min	β+ (95%), EC (5%)	Cardiology	▪ [^82^Rb]RbCl—myocardial perfusion imaging [31].
^86^Y	14.7 h	β+ (32%), EC (68%)	Oncology	▪ [^86^Y]Y-DOTA-Phe^1^-Tyr^3^-Octreotide—PET surrogate for ^90^Y therapy, enabling dosimetry and biodistribution assessment in NETs [32].
^89^Zr	78.4 h	β+ (23%), EC (77%)	Oncology	▪ [^89^Zr]trastuzumab—HER2-positive breast cancer [33]. ▪ [_89_Zr]Zr-DFO-onartuzumab—PET surrogate for predicting response to Met-targeted radioligand therapy in pancreatic cancer [34].

PET has proven to be an exceptionally powerful technique for diagnosing and monitoring the progression of diseases such as neurological disorders, cancer, cardiovascular disorders, and infections [35]. Moreover, its use contributes to guiding therapeutic interventions, such as radiotherapy, drug administration and surgery, by monitoring the effectiveness of these treatments. Beyond its diagnostic applications, PET is crucial in drug research and development, offering rapid in vivo data access. In pre-clinical phases, it helps determine whether a biological target is associated with a specific disease, evaluates the biological parameters of drug candidates—such as target engagement, nonspecific binding, blood–brain barrier (BBB) penetration, and metabolism—to assess their suitability for treating a given condition, and provides valuable insights into the drug’s mechanism of action. In clinical trials, PET aids to establish dose ranges with small volunteer groups, thus accelerating drug development, reducing adverse effects, and minimizing trial sizes [36].

Based on all these considerations, it is reasonable to assume that a radiotracer must meet certain key requirements to be effective. These include high specificity and selectivity for the target, as well as sub-nanomolar or nanomolar affinity for the physiological target to avoid interfering with normal biological functions.

In this context, paradigms already established in drug discovery have progressively been translated into radiopharmaceutical development, including allosteric targeting. Thus, alongside classical receptor targeting through orthosteric sites, namely the binding sites of endogenous ligands, allosteric modulators (AMs), by binding to topographically distinct allosteric pockets, provide a nuanced control over receptor activity and agonist affinity. In the presence of an agonist, they can enhance (PAMs), reduce (NAMs), or leave unchanged (NALs) its potency or affinity. This may result in improved selectivity for different receptor subtypes and tissues, reduced receptor desensitization, and better preservation of the temporal and spatial fidelity of agonist signaling, while the “ceiling effect”, dependent on the cooperativity between the two ligands, may contribute to reduced toxicity and lower risk of overdose. These advantages make allosteric targeting promising not only for therapeutic agents but also for radiopharmaceuticals.

Additionally, the radiotracer must exhibit low toxicity, good in vivo stability, rapid plasma clearance, and low plasma protein binding, as well as neutral and hydrophilic properties to enhance elimination and minimize the effective radiation dose. This has contributed to a greater awareness of the properties that carrier molecules should possess according to both the selected radionuclide and the intended imaging application. In particular, small molecules, owing to their rapid kinetics, favorable BBB permeability, and generally lower molecular weight, are more suitable for CNS imaging, whereas biomolecules (e.g., peptides, proteins, and antibodies) are often preferred for peripheral imaging applications because of their slower pharmacokinetics and limited BBB penetration. Consistently, radionuclide selection must align with the physicochemical and pharmacokinetic properties of the vector: short-lived radionuclides such as ^11^C and ^18^F are generally better suited for small molecules, whereas larger molecular constructs with longer biological kinetics typically require longer-lived radionuclides such as ^64^Cu and ^89^Zr.

Thus, the success of PET technology largely depends on the development of safe, highly sensitive and specific radiotracers targeting clinically relevant biomarkers. These include the G protein-coupled receptor (GPCR) superfamily that comprises over 800 proteins encoded by the human genome, thus representing the largest and most ubiquitous family of transmembrane receptors that reside on cell surfaces [37]. These receptors function as highly adaptable membrane sensors, effectively serving as “message inboxes.” They can detect a wide range of physicochemical stimuli, including ions, small organic molecules (such as odorants, vitamins, and neurotransmitters), photons, hormones, growth factors, lipids (like sterols and fatty acids), peptides, and proteins (including glycoproteins and chemokines). By doing so, they translate extracellular signals into intracellular responses across the plasma membrane. When GPCRs are activated intracellularly by the binding of a specific signaling molecule or “agonist”, they trigger a cascade of signals mediated by heterotrimeric G proteins (G_s_, G_i/o_, G_q/11_, and G_12/13_), arrestins, and various downstream effectors [38]. G proteins control the activity of enzymes and channels, leading to changes in the levels of second messengers within cells and, consequently, influencing their functional activities. These processes operate through pathways involving protein kinases, modifications in the phosphorylation or activity of proteins, ion channel regulation, low molecular weight GTP-binding proteins, and shifts in gene expression. By employing these diverse signaling mechanisms, GPCRs govern a broad spectrum of cellular functions, ranging from fundamental aspects of cell biology—such as metabolism, growth, apoptosis, and migration—to specialized cellular responses, including muscle contraction or relaxation, glandular and epithelial secretion, and modifications in neuronal activity.

Consequently, GPCR signaling is pivotal in various pathological processes, including the regulation of the immune system, inflammatory responses, mood and behavior modulation, and the maintenance of homeostasis. This makes GPCRs the major pharmacological target, with approximately 35% (~700) of all drugs approved by the US Food and Drug Administration (FDA) targeting this receptor family [39]. Extensive analyses of all clinical trial candidates mapped onto the GPCR superfamily tree identified roughly 321 agents, 20% of which target 66 potentially novel GPCRs with no approved drugs to date [37].

For the longest time, drugs targeting GPCRs have generally been developed without the support of high-resolution structural knowledge, which indeed are immensely valuable for providing a wide range of templates for a rational drug design [40]. Furthermore, recent landmarks in cryo-electron microscopy (cryo-EM), which led to the characterization of more than 500 molecular structures, have given a strong impetus to improve our understanding of GPCR structural biology [41].

The long track record of progress in the structural biology and pharmacology of GPCRs, along with rapid advances in computational techniques, has been the keystone of the success in the GPCR research area in the past few decades. This progress has also significantly contributed to enriching the radiopharmaceutical landscape, highlighting the huge potential of GPCRs as molecular targets for non-invasive PET imaging across a wide range of diseases. In fact, as biomarkers, GPCRs can be studied on circulating cells, such as tumor or fetal cells, within exosomes found in blood or urine, or as cell surface proteins detectable through specialized probes. These methods have the potential to enhance personalized and precision medicine approaches for GPCR-based therapies by identifying individuals or patient groups expressing specific GPCRs that can be targeted with appropriate therapeutic agents, with treatment responses monitored through biomarker analysis [42].

This review highlights the potential of GPCRs as molecular targets for non-invasive imaging via PET, as they are central to numerous physiological processes and are involved in a wide range of diseases. We selected the most innovative patents from 2020 to 2025 that paved the way to radiolabeled GPCR probes, enabling the visualization and quantification of these receptors in vivo, and significantly improving our understanding of their role in health and disease. We classified the patented radiotracers according to their diagnostic applications, grouping them by target pathology. Recurrent structural motifs across the analyzed compounds are highlighted and discussed alongside their biological data.

## 2. Landmark Patents (2020–2025): Classification by Disease Area and GPCR Biomarker

### 2.1. Oncology

GPCRs are now being used as promising biomarkers in oncology, aiding in early diagnosis, staging and restaging, monitoring treatment response, and radiotherapy planning.

While the question of whether GPCR and G protein mutations may drive cancer progression remains debated [43], GPCR activity and post-receptor signaling events greatly contribute to the control of key cellular processes associated with the hallmarks of cancer, such as proliferation, metabolism, programmed cell death, ion and nutrient transport, and migratory behavior [44]. Moreover, substantial evidence supports aberrant GPCR expression across various cancer types. For instance, tumors of the neuroendocrine system frequently display a high prevalence of the serotonin 5-HT_4_ receptor, the glucagon-like peptide-1 receptor (GLP-1R), and the melanocortin-2 receptor (MC2R), whereas lysophosphatidic acid receptor 5 (LPA_5_) and the chemokine CCR6 receptor are predominantly expressed in pancreatic ductal adenocarcinoma. Beyond tumor cells, GPCRs are also expressed by multiple cell types within the tumor microenvironment, including stromal (fibroblast), vascular, immune, and inflammatory cells.

In this context, a study employing an unbiased (GPCRomic) approach systematically identified and quantified GPCR expression across multiple cancer cell types, revealing an unexpectedly broad and heterogeneous GPCR landscape. Notably, in each tumor type a common core set of GPCRs was identified, with several cancer cell types displaying expression of more than 150 distinct receptors, including those subsets detected at relatively high levels. These findings support the concept of tumor-specific “GPCR signatures,” whereby individual GPCRs or defined receptor patterns may serve as novel diagnostic biomarkers and/or therapeutic targets [45].

Although further validation is needed to understand how GPCRs may contribute to the malignant phenotype and may represent therapeutic targets, this comprehensive profiling underscores the extraordinary potential of GPCR expression patterns as a rich and still largely untapped source for PET imaging in oncology.

#### 2.1.1. Somatostatin Receptors (SSTRs)

Somatostatin (also known as growth hormone-inhibiting hormone, GHIH) is an endogenous peptide that exerts inhibitory effects on virtually all endocrine and exocrine secretions. It is produced by pancreatic δ-cells and inhibits the release of insulin and glucagon; furthermore, it is generated in the hypothalamus to inhibit the release of growth hormone (GH), adrenocorticotropic hormone (ACTH), prolactin and thyroid-stimulating hormones (TSH) from the anterior pituitary. Somatostatin derives from the 116-amino acid precursor preprosomatostatin, which undergoes proteolytic cleavage to prosomastatin that is further processed into two active isoforms: somatostatin-14 (SST-14) and somatostatin-28 (SST-28). Their effects are mediated by five GPCRs (SSTR1–5), which show distinct tissue distributions [46,47]. SSTR1 is abundant in the jejunum and stomach; SSTR2 in cerebrum, pituitary, pancreas, intestines and kidney; SSTR3 in the brain and testis; SSTR4 in fetal and adult brain and lungs; and SSTR5 in the brain, pituitary gland, pancreatic α- and γ-cells as well as in the gastrointestinal tract. Importantly, SSTRs are overexpressed in several tumors and endocrine disorders, such as acromegaly, making them key targets for diagnostic imaging.

In 1994, the first peptide-based radiopharmaceutical, ^111^In-pentetreotide, was approved by the FDA under the name Octreoscan^®^ for the diagnosis of NETs and certain non-NETs expressing SSTRs. In this compound, the selective SSTR2 agonist octreotide is conjugated to indium-111 through the chelator diethylenetriaminepentaacetic acid (DTPA), leading to ^111^In-DTPA-octreotide, which has long represented the standard for SSTR scintigraphy [48].

Since then, a major leap forward has occurred with the introduction of ^68^Ga-labeled somatostatin analogs for PET, offering improved sensitivity [49]. Tracers currently used in clinical practice include [^68^Ga-DOTA^0^-Tyr^3^]octreotide, (^68^Ga-DOTA-TOC, ^68^Ga-edotreotide), [^68^Ga-DOTA^0^-1NaI^3^]octreotide (^68^Ga-DOTA-NOC) and [^68^Ga-DOTA^0^-Tyr^3^]octreotate (^68^Ga-DOTA-TATE). All listed ^68^Ga-DOTA-peptides show higher affinity for SSTR2 than ^111^In-DTPA-octreotide, with ^68^Ga-DOTA-TATE displaying an affinity for SSTR2 approximately one order of magnitude higher than that of the other ^68^Ga-DOTA-peptides. Notably, not all these tracers are fully selective for a single SSTR subtype: for example, ^68^Ga-DOTA-NOC also shows high affinity for SSTR5 and, to a lesser extent, for SSTR3, while ^68^Ga-DOTA-TOC displays some affinity for SSTR5, whereas ^68^Ga-DOTA-TATE is highly selective for SSTR2 [25,50]. Overall, these agents provide superior spatial resolution and more favorable pharmacokinetics compared with conventional SSTR scintigraphy.

More recently, the need for improved lesion targeting, particularly in tumors with low SSTR expression, has fueled growing interest in SSTR antagonists as alternatives to classical agonist peptide vectors. These compounds were found to recognize a larger number of receptor binding sites, frequently resulting in higher tumor uptake and improved imaging performance, thereby partially challenging the previous assumption associated with agonists that efficient receptor internalization was the main determinant of effective SSTR-targeted imaging. Among them, JR11 has emerged as one of the most promising candidates for both diagnostic imaging and radionuclide therapy [25,51].

**Figure 2:**
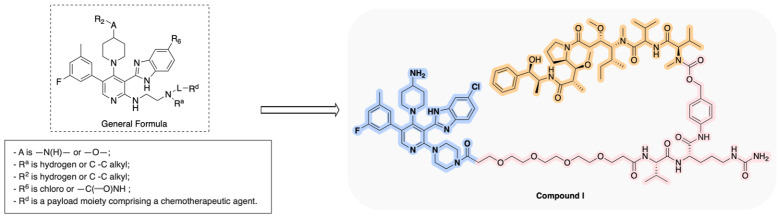
General formula of the non-peptide SSTR2 ligands based on a 4-piperidinyl-3-benzimidazole-6-arylpyridine scaffold (**left**) and representative structure of Compound **1** (**right**), comprising the SSTR2-targeting moiety (blue), linker (pink), and cytotoxic payload monomethyl auristatin E (orange) [52].

The search for optimized SSTR2-targeting ligands remains highly relevant. In 2024, a patent application by Crinetics Pharmaceuticals, Inc. proposed a new platform of small molecule drug conjugates (SMDCs) for selective cancer therapeutics or diagnostics [52]. These non-peptide SST2R ligands, built on a 4-piperidinyl-3-benzimidazole-6-arylpyridine scaffold (general formula, Figure 2), can be conjugated via spacer and/or linker moieties to suitable drug cargos or payloads, including not only chemotherapeutic agents but also radionuclides, thereby opening opportunities for PET imaging and theranostic applications. The central concept is to deliver functional agents that, on their own, exhibit limited selectivity, but which, when conjugated to an SSTR2 ligand, can be selectively directed to cancer cells, thus enhancing their therapeutic window.

To assess the biological activity of the SMDCs of the present invention, the compounds were first evaluated in CHO-K1 cells expressing SSTR2, where they consistently reduced intracellular cAMP levels in the low-nanomolar/subnanomolar range, in line with an agonistic profile. Internalization assays highlighted the impact of linker selection on receptor-mediated uptake, while antiproliferative effects were evaluated in NCI-H524 small cell lung cancer cells. However, subsequent in vivo investigations focused primarily on the indium-111 (^111^In)–radiolabeled form of Compound **1** (Figure 2), consisting of the cytotoxic payload monomethyl auristatin E (orange), a cathepsin B-cleavable/self-immolative linker system designed to enable intracellular payload release (pink), and the SSTR2-targeting ligand (blue). The biodistribution of [^111^In] Compound **1** was evaluated in female Swiss nude mice bearing xenografts of the SSTR2-positive rat pancreatic tumor cell line AR42J, showing high and sustained tumor uptake in a receptor-specific manner, with competition studies confirming selectivity. Non-specific uptake was limited to the kidneys, consistent with renal excretion as the main elimination pathway.

While the document refers to potential PET applications of the SMDCs, it does not provide experimental validation to support this aspect.

In the same year, Clarity Pharmaceuticals claimed the use of dimeric SSTR2-targeting radiopharmaceuticals based on a sarcophagine-chelated ^64^Cu complex bearing two SSTR2-binding moieties (e.g., octreotate), with preliminary biodistribution studies suggesting enhanced tumor retention compared with monomeric counterparts. The patent additionally proposed the sarcophagine scaffold as a strategy to improve radiometal stability and reduce radiolysis-related off-target damage, potentially supporting more efficient imaging and therapeutic applications in SSTR2-expressing cancers [53].

Along the evolution of peptide-based SSTR2 radiopharmaceuticals, in 2025 RayzeBio, Inc. described a broad series of macrocyclic peptides and conjugates designed for SSTR2 targeting and compatible with both diagnostic (e.g., ^68^Ga, ^64^Cu) and therapeutic radionuclides (e.g., ^225^Ac, ^177^Lu). The disclosed compounds showed SSTR2 affinity in the nanomolar/subnanomolar range and were proposed as highly modular peptide-based platforms capable of preserving receptor-targeting properties despite the conformational constraints introduced by amide-bond cyclization [54].

Another patent aimed to overcome some of the limitations associated with conventional SSTR2-targeting agents such as DOTATATE through a “tripartite” design integrating an SSTR2-binding moiety, an effector/radionuclide unit, and a fatty acid moiety intended to modulate pharmacokinetic behavior [55]. According to the inventors, this platform yielded compounds with higher SSTR2 affinity, with the most active derivatives displaying >8-fold lower IC_50_ values relative to DOTATATE, as well as increased tumor uptake while maintaining rapid systemic clearance in vivo. These properties may potentially enable a broader therapeutic window and more efficient tumor-targeted imaging and therapy, as preliminarily supported by biodistribution, imaging, and in vivo efficacy studies.

In recent years, the scientific community has increasingly focused on other SSTR subtypes expressed in certain tumors, with selective SSTR3-targeting ligands emerging as promising derivatives. SSTR3 is overexpressed in several malignancies, including gastroenteropancreatic neuroendocrine tumors (GEP-NETs), pituitary adenomas, and various hematological cancers such as sarcomas, myelomas, lymphomas, and leukemias. Moreover, SSTR3 expression has been reported in a wide range of solid tumors, including those of the pancreas, colon, breast, prostate, ovary, liver, kidneys, and lungs. Notably, SSTR3 is the only somatostatin receptor associated with p53-mediated apoptosis and cell cycle arrest, and it displays significantly higher internalization rates compared to other subtypes. These distinctive features, combined with its widespread expression in tumors, make SSTR3 a highly promising target for both diagnostic and therapeutic applications in oncology [56].

**Figure 3:**
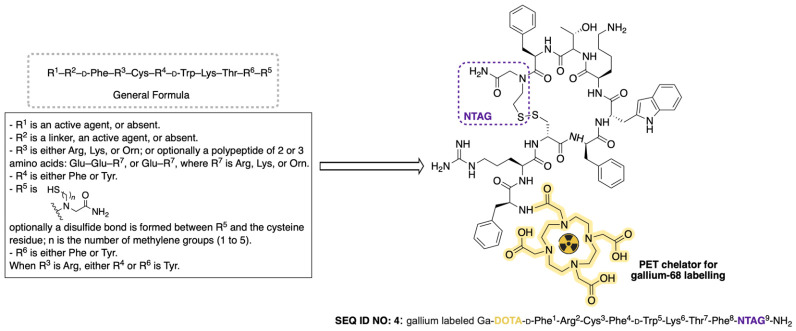
General structure of the conformationally constrained somatostatin analogs targeting SSTR3 (**left**) and representative structure of SEQ ID NO: 4 (**right**), illustrating the cyclic peptide scaffold with the characteristic NTAG moiety (purple) and its conjugation with a DOTA chelator for radiometal labeling (yellow) [57].

Accordingly, the May 2024 patent by Starget Pharma Ltd. specifically focuses on this receptor subtype, reporting a novel class of 28 conformationally constrained somatostatin analogs with high affinity and selectivity for SSTR3 [57]. The general structure of these compounds (R^1^–R^2^–D-Phe–R^3^–Cys–R^4^–D-Trp–Lys–Thr–R^6^–R^5^) consists of a sequence of amino acids interspersed with various R groups, strategically arranged to enhance receptor binding, subtype selectivity, and pharmacokinetic performance (general formula, Figure 3). A disulfide bridge between the cysteine residue and R^5^ group, consisting of an *N*-thioalkyl-glycine (NTAG), confers conformational rigidity and improved metabolic stability. The scaffold also supports *N*- or *C*-terminal conjugation with functional moieties such as radiometal chelators (e.g., DOTA), linkers, or bioactive agents, providing a versatile platform for the development of somatostatin analogs for both diagnostic and therapeutic purposes.

Among the tested compounds, SEQ ID NO: 4 (Figure 3) fully exemplified this dual potential, proving to be a nanomolar ligand with high selectivity for human SSTR3 and significantly lower affinity for the other SSTR subtypes. An additional set of pharmacological assays evaluated its potential off-target interactions, revealing no detectable agonistic or antagonistic activity across a panel of 167 human GPCRs. Furthermore, successful radiolabeling with ^68^Ga confirmed its suitability for PET imaging applications. Altogether, these findings highlight the superiority of this compound in fulfilling the key requirements for an SSTR3-selective radioligand. SEQ ID NO: 4 also demonstrated therapeutic relevance, supporting its classification as a theranostic candidate. Comparative studies in *h*SSTR3-transfected CHO cells showed that SEQ ID NO: 4 induces strong β-arrestin–mediated receptor internalization. Although it shares an EC_50_ comparable to the native hormone SRIF-14, SEQ ID NO: 4 elicited a β-arrestin response twice as strong, identifying it as a novel (first time known) superagonist of *h*SSTR3.

In vivo PET-CT imaging was performed following intravenous injection of SEQ ID NO: 4, with dynamic scans acquired for up to 240 min in mice and 270 min in rats. Biodistribution analysis revealed substantial tumor uptake in both models, with a favorable tumor-to-kidney ratio of approximately 50% observed in rats.

To further assess translational potential, a first-in-human PET-CT scan was conducted in a 37-year-old male patient with Desmoplastic Small Round Cell Tumor (DSRCT) known to express SSTR3. Imaging was performed at 30 and 120 min post-injection (∼600 MBq). The tracer showed rapid blood clearance and renal excretion, along with clear and specific tumor uptake. The PET signal was consistent with SSTR3 expression and aligned with the patient’s clinical prognosis. Importantly, the biodistribution confirmed the compound’s high selectivity for SSTR3, with no uptake in off-target organs and no evidence of unexpected toxicity, supporting both its diagnostic value and safety profile.

#### 2.1.2. Cholecystokinin 2 Receptor (CCK2R)

**Figure 4:**
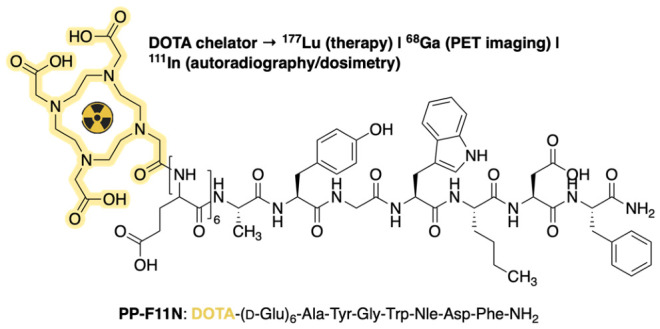
Structure of PP-F11N and its conjugation to a DOTA chelator enabling radiolabeling with different radionuclides (^177^Lu, ^68^Ga, ^111^In), each evaluated for distinct diagnostic or therapeutic applications [58,59,60,61].

The cholecystokinin 2 receptor (CCK2R, also known as cholecystokinin B receptor or CCKBR), a member of the GPCR family, has emerged as an attractive molecular target for both imaging and therapy in oncology, owing to its overexpression in several malignancies, including medullary thyroid cancer (MTC), small-cell lung cancer (SCLC), gastrointestinal stromal tumors (GIST), gliomas, as well as colorectal (CRC), breast (BC), and ovarian cancers. CCK2R is physiologically activated by gastrin, an endogenous linear peptide hormone initially encoded as pre-progastrin. Following post-translational enzymatic cleavage, progastrin is processed by G cells of the duodenum and pyloric antrum into mature gastrin, which is secreted into the bloodstream. In humans, gastrin occurs in different molecular forms, primarily big-gastrin (G-34), little-gastrin (G-17), and mini-gastrin. These peptides share a conserved C-terminal amino acid motif that enables high-affinity binding to CCK2R. On this basis, several studies have investigated radiolabeled gastrin analogues for molecular imaging and peptide receptor radionuclide therapy (PRRT), including the mini-gastrin derivative PP-F11N (DOTA-(D-Glu)_6_-Ala-Tyr-Gly-Trp-Nle-Asp-Phe-NH_2_), where the DOTA chelator stably coordinates radionuclides such as ^177^Lu (therapy) and ^68^Ga (PET imaging) (Figure 4) [58].

It is well established that high and sustained tumor uptake of a radiopharmaceutical directly depends on the expression level of its molecular target. This is particularly relevant for CCK2R, where, in some indications, receptor prevalence in tumor tissues has been reported to be low, with only 10–20% of patients exhibiting sufficient expression levels to enable effective PRRT. Moreover, the assessment of CCK2R expression by standard techniques, such as immunohistochemistry (IHC) or autoradiography, remains challenging, likely due to the limited selectivity and specificity of available anti-CCK2R antibodies.

To address this limitation, the Paul Scherrer Institute patented a biomarker-driven strategy demonstrating a strong correlation between CCKBR mRNA levels, receptor protein abundance, and radioligand uptake [59]. On this basis, CCKBR mRNA was proposed as a robust predictive biomarker for patient stratification in therapeutic or imaging procedures involving radiolabeled gastrin analogues targeting CCK2R.

Importantly, this patent does not introduce a novel targeting vector but builds upon the previously claimed mini-gastrin derivative PP-F11N [60], which had already demonstrated favorable properties, including high stability, suitable biodistribution, and reduced renal uptake when labeled with ^177^Lu.

Moreover, the patent published in August 2023 [61] discloses ^68^Ga-PP-F11N (Figure 4) as a specific PET imaging agent for CCKBR-positive tumors and reports a biodistribution profile closely resembling that of ^177^Lu-PP-F11N, thereby supporting its use in pre-selecting patients likely to benefit from CCK2R-targeted radionuclide therapy. Thus, in WO2023051897, PP-F11N was investigated as a case study to illustrate a biomarker-guided clinical workflow integrating CCKBR mRNA quantification in tumor biopsies to identify CCK2R-positive cases, PET-based confirmation of receptor expression using ^68^Ga-PP-F11N, and subsequent therapy with ^177^Lu-PP-F11N (Figure 4) in selected patients, with ^111^In-PP-F11N reserved for preclinical dosimetry. This integrated approach provides quantitative cut-off values for clinical decision-making and underscores the translational relevance of mRNA-based patient selection for CCK2R-targeted PET imaging and PRRT, while consolidating PP-F11N as a clinical reference ligand [59].

The PP-F11N scaffold was also the subject of recent patents by the groups of Béhé, Grob, Mindt, Schibli, and co-workers, mainly aimed at improving metabolic stability and tumor-to-kidney uptake ratios through innovative peptide stabilization strategies [62,63]. In particular, the disclosed mono- and multi-triazolominigastrins featured structurally optimized peptide backbones, linker regions, and *C*-terminal moieties, including the replacement of selected peptide amide bonds with 1,2,3-triazole bioisosteres to enhance metabolic stability while preserving receptor recognition. These analogs demonstrated high receptor-specific internalization, favorable IC_50_ values, sufficient plasma stability, and improved in vivo tumor-targeting properties compared with reference minigastrin derivatives, further supporting their potential use as diagnostic and therapeutic radiopharmaceuticals for the targeting of CCK2R-positive neoplasms.

Later, WO2024061483 described shortened tetrapeptidic minigastrin-derived ligands designed around a minimal CCK2R-binding pharmacophore. Several analogs retained favorable CCK2R affinity, which was also preserved after functionalization with chelators, linkers, or SiFA (silicon-fluoride acceptor) moieties for ^18^F-labeling, further supporting the versatility of these reduced peptide scaffolds [64].

Although the core of CCK2R-radioligand development is still strongly dominated by minigastrin-derived peptides and by strategies aimed at counteracting their rapid in vivo degradation, the field is also progressively moving toward non-peptidic approaches. Indeed, despite their less advanced clinical translation, recent studies have highlighted the potential advantages of CCK2R antagonists over peptide agonists, including reduced receptor activation-related side effects (e.g., gastrointestinal discomfort, transient cardiovascular changes, and systemic receptor activation) and the possibility of accessing a larger number of receptor binding sites, potentially leading to higher tumor uptake [65]. In this context, the non-peptidic CCK2R antagonist Z-360 emerged as a promising scaffold for PET imaging and radionuclide therapy, as preclinical studies with ^111^In-, ^68^Ga-, and ^177^Lu-labeled derivatives demonstrated preserved CCK2R affinity, selective targeting of CCK2R-positive tumors, and favorable pharmacokinetic profiles [66].

#### 2.1.3. Dual-Receptor Targeting Strategy: SSTR2 and CCK2R

SSTRs can be co-expressed with other targets in heterogeneous tumors. For instance, aggressive NETs frequently express both SSTR2 and CCK2R. A ligand capable of engaging both receptors may improve imaging sensitivity and therapeutic efficacy compared to agents targeting a single receptor. Moreover, a modular design may support broader tumor uptake—addressing SSTR2-positive, CCK2R-positive, or double-positive cells—and promote enhanced retention of the radiopharmaceutical through multivalent binding mechanisms.

Dual-targeted radiopharmaceuticals addressing both SSTR2 and CCK2R represent the core concept of a patent dated 2024 [67]. These novel hybrid compounds hold promise for PET imaging of SSTR2- and CCK2R-positive tumors as well as for targeted radionuclide therapy of receptor-expressing lesions.

**Figure 5:**
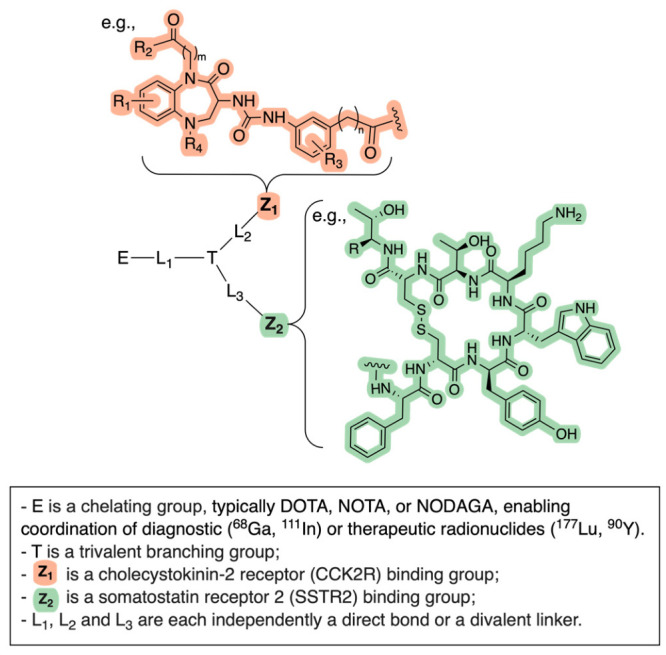
General formula of dual SSTR2- and CCK2R-targeting radioligands [67].

Figure 5 illustrates the modular design of a trifunctional molecule, where a trivalent branching unit (T) connects three domains: *(i)* a chelating group (E), *(ii)* a CCK2R-binding motif (Z_1_), and *(iii)* an SSTR2-binding motif (Z_2_), each joined by flexible spacers (L_1_–L_3_). The SSTR2-targeting unit is typically a somatostatin-derived peptide (e.g., octreotide or octreotate), while the CCK2R-binding domain is often derived from a modified cholecystokinin fragment. These pharmacophores are joined via a bifunctional linker, which frequently incorporates a metal-chelating group, such as DOTA or macrocyclic chelators, thereby enabling stable incorporation of diagnostic (such as ^68^Ga or ^111^In) or therapeutic (such as ^177^Lu or ^90^Y) radiometals. Variants of the linker (L_1_–L_3_), including polyethylene glycol (PEG) chains, neutral amino acid bridges (e.g., glycine or serine), or other bifunctional moieties, have been employed to modulate spatial arrangement and optimize dual receptor binding.

The patent further covers pharmaceutical formulations comprising any of the described compounds or their radiolabeled derivatives, in combination with pharmaceutically acceptable excipients. These formulations may include, for instance, sterile injectable solutions suitable for intravenous administration of the dual-target radiopharmaceutical. The scope also extends to kit formulations, such as lyophilized ligands intended to be combined with a radionuclide immediately prior to administration, and to methods of treatment or diagnosis involving the administration of any of the radiolabeled compounds described.

It should be noted, however, that while the patent outlines the potential use of these compounds in PET imaging, it does not present experimental evidence to support this application.

#### 2.1.4. CXC Chemokine Receptor Type 4 (CXCR4)

CXCR4 is a GPCR expressed on monocytes, B cells, naïve T cells, neutrophils and eosinophils. It is involved in multiple physiological functions and interacts with its natural ligand chemokine ligand 12 (CXCL12), also known as stromal cell-derived factor 1 (SDF-1) [68]. Over the past decades, the CXCR4–CXCL12 signaling axis has been the focus of extensive research because of its key contribution to the onset and progression of several hard-to-treat conditions, including HIV infection, inflammatory disorders, and metastatic cancers such as breast, gastric, and non-small cell lung cancer [69]. This pathway promotes angiogenesis and mobilization of bone marrow-derived myeloid cells, facilitating tumor recurrence and metastasis and contributing to resistance against both conventional and targeted treatments. Approaches aimed at blocking CXCR4/CXCL12-mediated chemotaxis—using antibodies, peptide-based inhibitors, or small-molecule antagonists—have shown promise in enhancing white blood cell mobilization while reducing metastasis by preventing tumor cell migration and their homing to secondary organs. Given its central role in cancer biology, CXCR4 has emerged as a compelling target for the development of both diagnostic imaging tools and therapeutic interventions.

**Figure 6:**
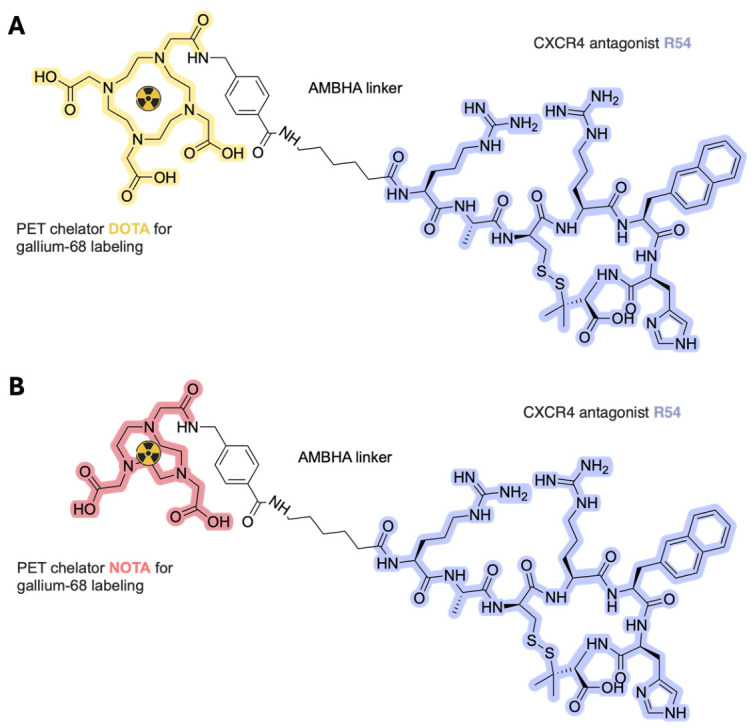
Structures of [^68^Ga]DOTA-AMBHA-R54 (**Panel A**) and [^68^Ga]NOTA-AMBHA-R54 (**Panel B**) [72].

A wide range of CXCR4-targeted PET tracers has been developed over the past years. While preclinical studies have yielded highly promising candidates with strong CXCR4 affinity and excellent targeting properties, the number of compounds that have advanced into clinical testing remains limited, and their translation has been hampered by unfavorable pharmacokinetics and/or suboptimal tumor uptake [70]. Nevertheless, ongoing efforts in CXCR4-targeted PET probe design and clinical validation are expected to overcome these challenges. Reflecting this trend, a 2023 patent from the Istituto Nazionale Tumori IRCCS—Fondazione G. Pascale drew on the highly specific CXCR4 antagonist R54 [71] as a structural template [72]. R54 is a cyclic heptapeptide (Ac-Arg-Ala-[D-Cys-Arg-Nal(2′)-His-Pen]-COOH) stabilized by a D-Cys^3^–Pen^7^ disulfide bridge. It exhibits high serum stability, nanomolar CXCR4 affinity (IC_50_ ≈ 1.5–20 nM depending on the assay), and potent antagonist activity [73]. Thus, the present invention relates to labeled R54 analogues designed as tracers for the selective targeting and imaging of human CXCR4-expressing cells, including those found in primary and secondary tumors, as well as in neoplastic and tumor-infiltrating immune cells. To this end, the *N*-terminus of R54 was modified by introducing 6-(4-(aminomethyl)benzamido)hexanoic acid (AMBHA) as a linker, while NOTA and DOTA were employed as chelators for radiometal labeling (Figure 6). AMBHA was selected to provide an appropriate linker length, maintaining distance between the metal chelator and the interaction site on the receptor. The resulting conjugates, NOTA-AMBHA-R54 and DOTA-AMBHA-R54, were radiolabeled with ^68^Ga and evaluated by in vitro and in vivo studies, which confirmed their potential for high-contrast CXCR4-targeted PET imaging. PET imaging and biodistribution studies were performed in athymic mice bearing CHO-*h*CXCR4 xenografts, with receptor expression confirmed by immunohistochemistry. Both [^68^Ga]DOTA-AMBHA-R54 and [^68^Ga]NOTA-AMBHA-R54 exhibited rapid clearance and negligible retention in non-target tissues, except for renal uptake. Consistent with its higher CXCR4 affinity, [^68^Ga]NOTA-AMBHA-R54 demonstrated superior tumor accumulation and higher tumor-to-background ratios than the DOTA analogue. Competition with excess unlabeled R54 reduced uptake to background levels, confirming the specificity of the binding. PET imaging confirmed efficient targeting of CXCR4-positive tumors by both tracers and further highlighted the advantage of the NOTA derivative, with SUV_max_ values exceeding expectations from biodistribution data, and tracer uptake strongly correlated with CXCR4 expression levels. Overall, both [^68^Ga]NOTA-AMBHA-R54 and [^68^Ga]DOTA-AMBHA-R54 proved suitable as CXCR4-targeted PET tracers, with the NOTA analogue offering clear advantages in terms of affinity, tumor uptake, clearance, and imaging contrast. Its favorable pharmacokinetic profile also supports its potential as a scaffold for future therapeutic applications.

**Figure 7:**
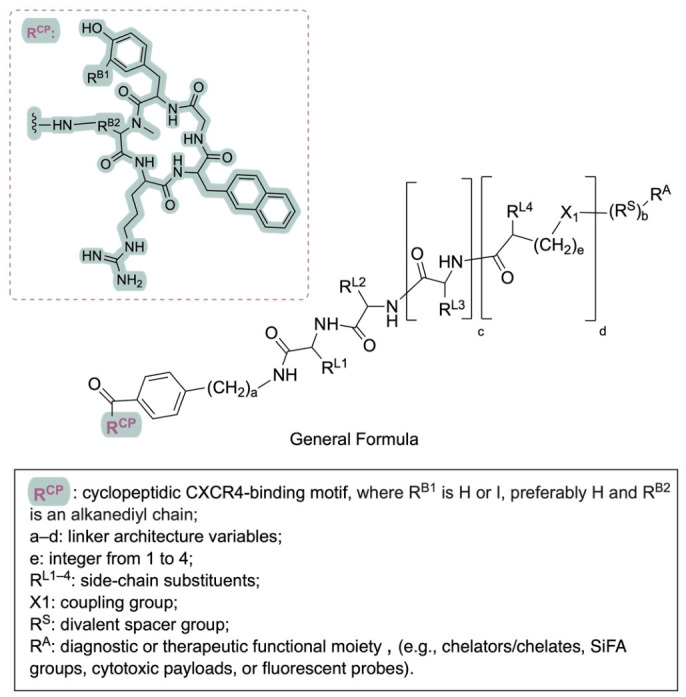
General formula of CXCR4-ligands disclosed in the patent [75].

In another patent, Hans-Jürgen Wester, Matthias Konrad, and Margret Schottelius disclosed a novel platform of modular cyclopeptidic CXCR4 ligands (general formula, Figure 7), in which the receptor-binding motif (*R*^CP^) was inspired by the potent CXCR4 antagonist FC-131 [74] and used as an anchor point for further structural optimization [75]. A key strength of these ligands lies in their high flexibility toward the introduction of different diagnostic or therapeutic functional units while preserving, or even enhancing, CXCR4 affinity. Indeed, in vitro studies evaluated receptor affinity, receptor-mediated internalization, and lipophilicity of the CXCR4 ligands through competitive binding assays on CXCR4-positive cells. Notably, the radiolabeled analogs retained high CXCR4 affinity and enhanced specific internalization even after functionalization with chelators or radionuclides (e.g., ^125^I, ^177^Lu, ^99m^Tc, ^18^F, or ^68^Ga). Additionally, these analogs demonstrated promising in vivo biodistribution and tumor-targeting performance, supporting their potential use for both molecular imaging and therapeutic applications, including endoradiotherapy.

#### 2.1.5. Neurokinin 1 Receptor (NK1R)

**Figure 8:**
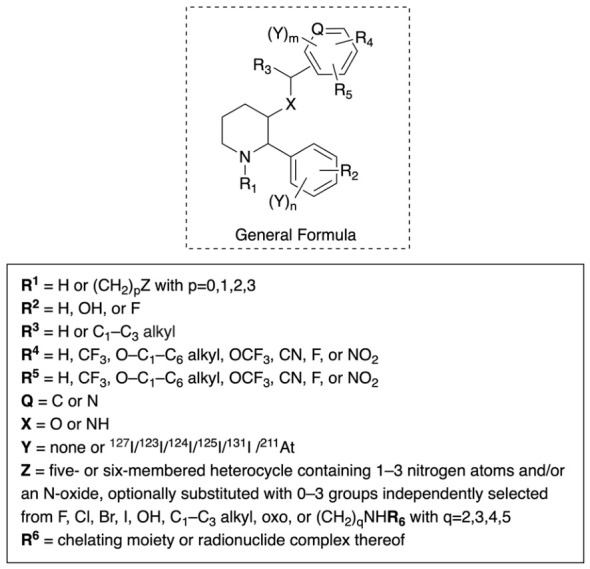
General formula of substituted 2-phenylpiperidine compounds [79].

The neurokinin 1 receptor (NK1R, also known as tachykinin 1 receptor) is a member of the tachykinin receptor subfamily of GPCRs and is widely expressed in both the central and peripheral nervous systems. Its principal endogenous ligand is substance P (SP), which regulates diverse physiological processes, including hematopoiesis, wound healing, microvascular permeability, neurogenic inflammation, leukocyte trafficking, and cell survival [76]. Importantly, NK1R expression is upregulated in multiple-cancer types, such as astrocytoma, melanoma, prostate cancer, glioma, retinoblastoma, leukemia, and tumors of the pancreas, larynx, colon, stomach, and breast. The SP/NK1R axis has been documented to contribute to tumor progression by promoting mitogenesis, angiogenesis, cell migration, and metastasis; accordingly, NK1R antagonists have been proposed as promising therapeutic agents in oncology. Among them, aprepitant (commercially known as EMEND or L-754,030) is particularly noteworthy. Approved for the treatment of chemotherapy-induced nausea and emesis, aprepitant has also shown antitumor properties, supporting its potential candidacy for future applications in prostate cancer therapy [77]. The structure of this small-molecule NK1R antagonist inspired several structure−activity relationship (SAR) programs aimed at improving its pharmacokinetic properties, which had hampered clinical development [78]. The derivative portfolio has also been expanded with novel aprepitant analogues functionalized with a DOTA chelator and radiolabeled with ^68^Ga or ^177^Lu, to further investigate their potential as radiopharmaceuticals. In line with the search for novel NK1R-targeting agents derived from small-molecule antagonists, a 2025 patent disclosed 54 newly substituted 2-phenylpiperidine derivatives (general formula shown in Figure 8), predominantly designed as radioiodinated analogues—mostly intended for therapeutic applications, except for ^124^I, which enables PET imaging—for the diagnosis, treatment, and/or prevention of cancer [79]. Within this chemical series, several analogues demonstrated low-nanomolar affinity for NK1R across different NK1R-positive cancer cell lines, including neuroblastoma, colorectal, and osteosarcoma models. Binding assays were consistently accompanied by competition tests, which confirmed receptor-mediated uptake and minimal non-specific binding, thereby supporting the selective accumulation of these compounds at target sites. Although development has so far been limited to binding and cellular studies without in vivo imaging evidence, these compounds bind NK1R specifically and competitively, reinforcing the value of this chemical platform for diagnostic and therapeutic development.

#### 2.1.6. Melanocortin Type 2 Receptor (MC2R)

**Figure 9:**
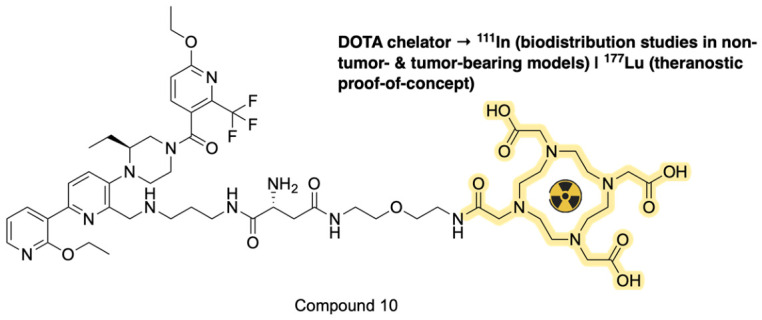
^111^In- and ^177^Lu-labeled radiocomplexes of a representative non-peptidic MC2R-targeting ligand disclosed in the patent, with the DOTA chelator highlighted in yellow [81].

MC2R, the smallest human GPCR, is exclusively activated by adrenocorticotropic hormone (ACTH) and is predominantly expressed in the adrenal cortex. ACTH-mediated activation of MC2R initiates cAMP-dependent signaling pathways that promote glucocorticoid biosynthesis, primarily cortisol. Altered MC2R activity has been associated with impaired adrenal function, resulting in adrenal insufficiency, which may lead to hypoglycemia, abnormal stress responses, and metabolic imbalances [80]. Owing to its tissue-specific expression and its involvement in cortisol-producing adrenal tumors, MC2R represents an attractive molecular target for selective imaging and radionuclide-based therapeutic approaches in adrenal malignancies, including adrenocortical carcinoma (ACC). Zhao et al. reported a series of non-peptidic MC2R-targeting ligands based on a substituted aryl–heteroaryl scaffold, frequently functionalized with a piperidinyl moiety and a linker-separated chelating group or radionuclide complex (e.g., ^111^In, ^115^In, ^67^Ga, ^68^Ga, ^177^Lu), including PET-relevant radiometals such as ^68^Ga, although the platform is primarily proposed within a theranostic framework [81]. Biodistribution studies in non-tumor-bearing rats mainly focused on ^111^In- and ^177^Lu-labeled radiocomplexes of Compound **10** (Figure 9), revealing high and prolonged uptake in the adrenal glands. Receptor specificity was confirmed by selective blocking experiments, demonstrating MC2R-mediated uptake with minimal to no accumulation in non-MC2R-expressing organs. Subsequent imaging studies in MC2R-positive tumor models using ^111^In–Compound **10** showed sustained and selective radiotracer accumulation in tumors as well as in adrenal glands, the only known site of endogenous MC2R expression. Specificity was confirmed by competition assays. Uptake in non-target organs, including blood, brain, and other tissues, was minimal, while renal and hepatic activity was transient and attributed to excretory pathways. Notably, ^177^Lu-labeled derivatives demonstrated proof-of-concept antitumor efficacy in MC2R-expressing xenograft models, supporting the feasibility of MC2R-directed theranostic approaches.

#### 2.1.7. Kisspeptin Receptor (KISS1R)

KISS1R, also known as GPR54, is a member of the GPCR family and is overexpressed in several cancers, including breast cancer, renal cell carcinoma, and liver cancer. Its endogenous ligand, kisspeptin (KP), is a neuropeptide hormone that stimulates the hypothalamo–pituitary–gonadal (HPG) axis and is derived from proteolytic processing of a 145-amino-acid precursor encoded by the KISS1 gene. Kisspeptin is initially produced as a 54-amino-acid peptide and can be further processed into shorter biologically active fragments, among which kisspeptin-10 (KP10) shows high affinity for KISS1R. KP10 triggers G_q/11_-mediated signaling, leading to phospholipase C activation and generation of inositol-1,4,5-triphosphate and diacylglycerol, which in turn induce intracellular calcium mobilization and MAPK pathway activation. This signaling cascade plays a key role in the modulation of the reproductive axis and endocrine function [82]. By regulating the release of gonadotropin-releasing hormone (GnRH) in the hypothalamus, this system drives pituitary gonadotropin secretion, which is essential for the onset of puberty and the maintenance of fertility [83].

**Figure 10:**
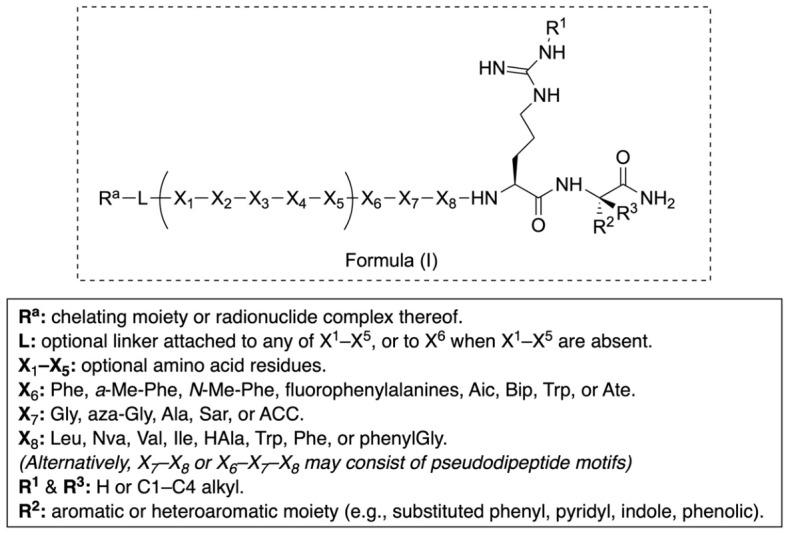
Simplified schematic of the kisspeptin-derived conjugate platform defined by Formula (I) disclosed in WO2024206577 [87].

Beyond its reproductive role, the kisspeptin system exerts additional non-canonical functions in a range of pathological conditions, notably in cancer progression and metastasis. Altered KISS1/KISS1R signaling was found to be implicated in multiple tumor types, including melanoma, prostate and endometrial carcinomas, leiomyomas and leiomyosarcomas, breast cancer, choriocarcinoma, as well as epithelial and stromal ovarian tumors. Reduced receptor expression has been associated with increased tumor aggressiveness, invasion, and distant metastasis in gastric cancer, whereas in breast cancer, KISS1R upregulation correlates with more aggressive disease and increased mortality risk [84,85]. Therefore, several kisspeptin peptide analogues have been developed to modulate kisspeptin signaling, effectively suppressing GnRH release and emerging as promising candidates for the treatment of hormone-dependent diseases, including prostate cancer. These analogues generally exhibit improved metabolic stability compared to native kisspeptins while retaining robust KISS1R agonist activity. In this context, the development of radiopharmaceuticals targeting KISS1R represents an attractive strategy for advancing cancer diagnosis and therapy [86]. For instance, Radionetics Oncology, Inc. has reported the development of a chemically versatile class of kisspeptin-derived conjugates described by the general Formula I (Figure 10). The disclosed conjugates feature extensive backbone and side-chain modifications, including non-natural amino acids, stereochemical inversions, and terminal functionalization. Importantly, this modular design enables conjugation to chelators or radionuclides, indicating a strategic focus on KISS1R-targeted diagnostic and therapeutic modalities [87]. Supporting the proposed use of KISS1R-targeted conjugates for PET imaging, the applicants report preferential KISS1R targeting, including 10- to 1000-fold higher affinity for KISS1R over non-target receptors, together with selective accumulation in KISS1R-expressing tumor tissues. Therefore, this platform-based patent outlines a versatile radiopharmaceutical strategy while leaving experimental PET validation to future studies.

#### 2.1.8. Neurotensin Receptor 1 (NTSR1)

**Figure 11:**
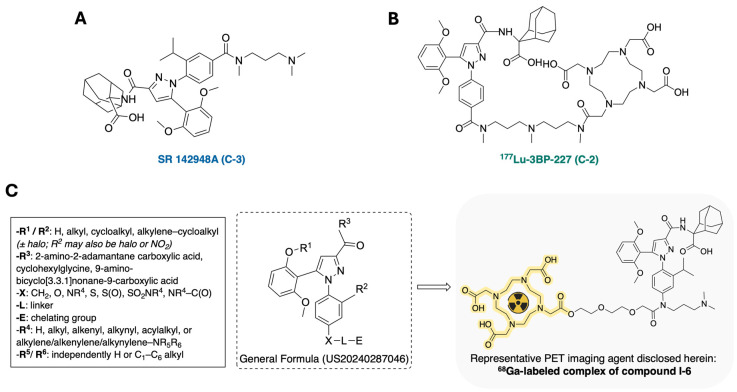
(**A**) Structure of SR142948A (C-**3**); (**B**) structure of ^177^Lu-3BP-227 (C-**2**); (**C**) general formula of the newly developed SR142948A-derived NTR1-targeting radioligands, with the DOTA chelator highlighted in yellow [96].

Neurotensin (NT) is an endogenous tridecapeptide with the sequence *p*Glu–Leu–Tyr–Glu–Asn–Lys–Pro–Arg–Arg–Pro–Tyr–Ile–Leu, whose primary biological activity resides in the highly conserved *C*-terminal fragment NT (8–13). NT functions as a neurotransmitter in CNS, where it is involved in the regulation of pain modulation, thermoregulation, feeding behavior, reward, and also acts as a hormone in the gastrointestinal tract. Its biological effects are mediated by three neurotensin receptor (NTSR) subtypes: the classical GPCRs NTSR1 and NTSR2, and the non-GPCR receptor NTSR3 (sortilin) [88,89]. NTSR1 is the main mediator of NT signaling, displaying effects in both the CNS and the periphery, whereas NTSR2 is predominantly expressed in the brain and NTSR3 displays a broader tissue distribution. Although NT biology primarily originates in the CNS and, together with its receptors, constitutes the neurotensinergic system, marked NTSR1 overexpression has been reported in several solid tumors, including colorectal and pancreatic cancers—most notably pancreatic ductal adenocarcinoma (PDAC)—as well as breast, prostate, and both small- and non-small-cell lung cancers [90,91,92]. Accumulating evidence since the early 2000s has linked NTSR1 overexpression to disease progression, highlighting this receptor as an attractive target for oncological imaging and theranostic strategies [93]. Accordingly, high-affinity small-molecule NTSR1 antagonists, such as SR142948A (C-**3**, Figure 11), have enabled the development of NTSR1-targeted radioligands for tumor imaging and therapy, with preclinical validation and initial clinical evaluation in pancreatic cancer patients (e.g., ^177^Lu-3BP-227 or C-**2**, Figure 11) [94,95]. However, prior derivatization strategies of SR142948A often resulted in reduced receptor binding affinity and compromised tumor-to-kidney and tumor-to-normal-organ ratios. These limitations have prompted the development of optimized SR142948A derivatives and next-generation radioligands targeting neurotensin receptors to achieve a wider therapeutic window for clinical use. Along these lines, in August 2023, analogues of SR142948A and their novel radioligand derivatives, based on the general formula depicted in Figure 11, were developed, including compounds intended for PET imaging [96]. Competitive radioligand binding assays showed that all compounds display high affinity for NTSR1, with IC_50_ values in the 0.01–1 nM range, indicating that linker architecture between the aromatic core and the chelator can unexpectedly influence receptor binding. Functional activity was evaluated by FLIPR calcium-mobilization assays in HEK293 cells expressing human NTSR1, where several compounds inhibited neurotensin-induced intracellular Ca^2+^ signaling more effectively than the reference compound C-**2**.

The patent further reports comprehensive in vivo biodistribution studies of ^177^Lu-labeled complexes in ASPC-1 pancreatic and HT-29 colorectal cancer xenograft models, revealing high and sustained tumor uptake, significantly improved tumor-to-normal organ ratios, and increased tumor AUC compared with the reference compound C-**2**, thereby highlighting the critical influence of linker design on the in vivo performance.

Importantly, PET imaging studies in ASPC-1 tumor-bearing mice using ^68^Ga-labeled complexes demonstrated excellent tumor-to-normal tissue contrast over 180 min, with analogue I-**6** emerging as the most promising candidate (Figure 11). These imaging data corroborated the biodistribution findings, thus supporting the use of these ligands for PET detection of NTSR1-positive tumors.

Finally, the patent concludes with in vivo efficacy studies of ^177^Lu- and ^225^Ac-labeled complexes in ASPC-1 tumor-bearing mice, which demonstrated a pronounced tumor growth inhibition for selected compounds, further underscoring their therapeutic potential.

A few months later in the same year, a patent filing from the University of North Carolina further explored the non-peptidic NTSR1-targeting scaffold derived from the antagonist SR142948A and modified through the introduction of crosslinked polyamines, leading to SR-CP-05-based agents [97]. In particular, the inventors observed that the ^64^Cu-labeled SR-CP-05 outperformed both peptide-based NT analogs and the previously reported ^64^Cu-3BP-227 (the ^64^Cu-labeled version of C-**2** in Figure 11), achieving a tumor uptake of 15.6% ID/g. Additionally, the newly developed SR-CP-05 ligand demonstrated high tumor-to-background contrast (>20 at 1 h post injection) together with minimal washout even after 48 h post injection. As observed for tumor uptake, the superior retention and contrast profile compared with peptide-based tracers further support the promise of SR-CP-05-based agents not only for imaging applications, but also for potential theranostic approaches in prostate cancer management.

#### 2.1.9. Glucagon-like Peptide-1 Receptor (GLP-1R)

GLP-1 receptor is a GPCR highly expressed on pancreatic β-cells and markedly overexpressed in insulinomas, gastroenteropancreatic neuroendocrine tumors arising from β-cell proliferation. Owing to this selective expression profile, GLP-1R has emerged as an attractive molecular target for β-cell mass (BCM) imaging. Physiologically, GLP-1R mediates the effects of glucagon-like peptide-1 (GLP-1), an incretin hormone produced mainly by enteroendocrine L cells of the intestine, pancreatic α-cells, and the CNS, where it contributes to glucose regulation, gastric emptying, and appetite control. These biological functions have made the GLP-1/GLP-1R axis highly relevant for the management of type 2 diabetes and obesity. Nevertheless, the direct use of native GLP-1 for imaging applications is severely limited by its extremely short plasma half-life, primarily due to rapid enzymatic degradation by dipeptidyl peptidase-4 (DPP-4), resulting in poor in vivo stability and limited suitability as a PET radiotracer [98]. To overcome the intrinsic instability of native GLP-1, research efforts progressively shifted toward the development of long-acting GLP-1 analogues. Among these, Exendin-4 has attracted considerable interest due to its therapeutic potential in diabetes management. This 39-amino-acid peptide, originally isolated from the salivary secretions of the Gila monster (*Heloderma suspectum*), shares substantial structural similarity with endogenous GLP-1 and reproduces many of its glucoregulatory effects through activation of GLP-1R [99,100].

Over the past few years, several radiolabeled derivatives of Exendin-4 have been investigated as potential agents for BCM monitoring and insulinoma imaging. However, many of these approaches have been limited by pronounced and persistent renal accumulation, which represents a major challenge for pancreatic imaging due to the anatomical proximity of the kidneys and surrounding abdominal tissues.

**Figure 12:**
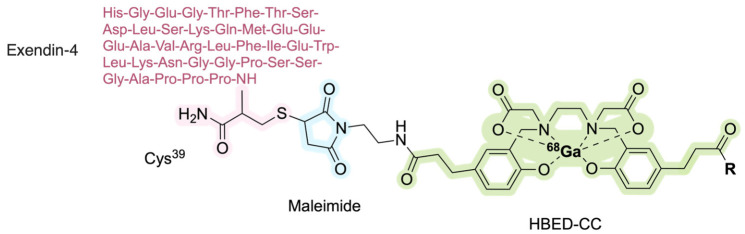
Structure of the ^68^Ga-labeled Exendin-4–based radiotracer ([^68^Ga]Ga-HBED-CC-MAL-Cys^39^-exendin-4) disclosed in patent [102].

The patent work by Lin Zhu and co-inventors fits within this context and describes a ^68^Ga-labeled Exendin-4–based radiotracer, ([^68^Ga]Ga-HBED-CC-MAL-Cys^39^-exendin-4), whose structure is reported in Figure 12. Among its most notable structural features is the incorporation of the bifunctional chelator *N*,*N*′-bis[2-hydroxy-5-(carboxyethyl)benzyl]ethylenediamine-*N*,*N*′-diacetic acid (HBED-CC), a highly efficient Ga(III)-complexing agent capable of forming thermodynamically stable ^68^Ga complexes under relatively mild labeling conditions (log*K*_GaL_ ≈ 38.5) [101]. In the disclosed structures, the HBED-CC scaffold is further functionalized with different R substituents, including dodecylamine, 4-(*p*-iodophenyl)butyric acid, and 4-(*p*-methylphenyl)butyric acid, likely introduced to modulate the pharmacokinetic behavior of the resulting radiotracers. According to the patent, these Exendin-4–based radiotracers displayed high affinity and specificity toward the GLP-1 receptor, together with elevated target uptake and prolonged circulation time, supporting its potential as a GLP-1R-targeted imaging agent for BCM monitoring in diabetes and for insulinoma localization [102].

### 2.2. CNS Diseases

Nowadays, PET scanning is a widely used imaging technique in the clinical neuroscience practice, as it greatly improves the understanding of the pathophysiology and treatment of CNS diseases, such as neurodegenerative disorders, including Alzheimer’s disease (AD), Parkinson’s disease (PD) and multiple sclerosis, as well as psychiatric conditions, including schizophrenia and depression.

PET imaging effectively detects cerebral biomarkers using contrast agents composed of radioactive molecules rationally designed to selectively bind to target receptors, transporters, or to be metabolized by specific enzymes. By measuring the spatial distribution and concentration of these imaging agents within the CNS, PET enables a detailed quantitative analysis of biomarker dynamics, providing valuable insights into diverse CNS functions, among them neurotransmission, metabolic pathways, and inflammatory responses [103,104].

The ideal biomarker must have a high sensitivity and specificity, be easy to measure, provide reproducible results and reflect disease progression. Circulating microRNAs (miRNAs) and extracellular microvesicles—known as exosomes—are an example of molecular biomarkers that may be useful in the diagnosis of some pathological CNS conditions. Among others, it is worth noting tau protein, a cerebrospinal fluid biomarker considered a diagnostic criterion for AD. Accordingly, PET tau imaging agents are thought to represent the amount and distribution of tau protein plaques in the brain, providing insights into tau-related changes [105]. Additionally, one of the most widely studied biomarkers for PET imaging of neuroinflammation—which, as we know, is a significant hallmark of CNS disorders—is the 18 kDa translocator protein (TSPO), that is uniformly expressed on activated cells of the myeloid lineage, reactive astrocytes, and endothelial cells. However, TSPO PET tracers suffer from variable binding affinity in human subjects due to a polymorphism in the *TSPO* gene.

Several pieces of evidence strongly suggest that GPCRs are excellent biomarkers for PET imaging of CNS diseases [106]. One of the key strengths of GPCRs is their high specificity for certain cell types and physiological processes, allowing PET imaging to precisely target CNS signaling pathways while minimizing off-target effects. Unlike static biomarkers (e.g., protein aggregates), GPCRs provide dynamic functional insights by visualizing real-time processes such as neurotransmitter activity and receptor signaling. Notably, GPCR activity often changes at an early stage of disease progression, even before structural damage occurs, making these receptors valuable for understanding disease onset and monitoring therapeutic responses.

Other advantages are the GPCR widespread expression across the CNS, enabling the study of various neurological conditions, and their well-characterized pharmacology as drug targets, which clearly facilitates the development of radiotracers.

#### 2.2.1. Metabotropic Glutamate Receptors 2 and 3 (mGluR2 and mGluR3)

L-Glutamate is the major excitatory neurotransmitter in the CNS and plays a crucial role in regulating several neurological functions. It acts through two major receptor families: ionotropic glutamate receptors (iGluRs) and metabotropic glutamate receptors (mGluRs) [107,108]. mGluRs (mGluR1–mGluR8), members of the GPCR superfamily, are classified into three groups. Among them, group II receptors—mGluR2 and mGluR3—are primarily located on presynaptic nerve terminals where they exert a negative feedback loop to the release of glutamate into the synapse [109]. Therefore, group II ligands, whether acting orthosterically or allosterically, relieve the inhibitory control on glutamate release, thereby enhancing glutamatergic signaling. This mechanism makes them promising candidates for the treatment of neurological disorders associated with dysregulated glutamatergic transmission, such as psychosis, mood disorders, AD, and cognitive or memory impairments [110]. For instance, Roche has investigated the mGluR2/3 antagonist RO4995819 in clinical trials as an adjunctive therapy for Major Depressive Disorder unresponsive to standard antidepressants and has also disclosed mGluR2/3 negative allosteric modulators (NAMs) as potential treatments for autism spectrum disorders.

**Figure 13:**
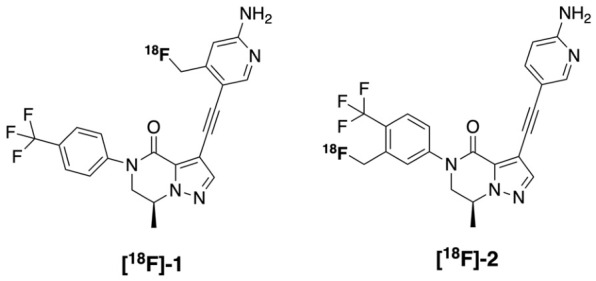
Structures of [^18^F]-**1** and [^18^F]-**2**, radiolabeled mGluR2/3 tracers disclosed by Janssen Pharmaceutica NV in 2020 [111].

In 2020, Janssen Pharmaceutica NV disclosed novel radiolabeled mGluR2/3 ligands for PET imaging and quantification of receptor expression in tissues [111]. Two fluorine-18 tracers, [^18^F]-**1** and [^18^F]-**2**, were developed on the 5-phenyl-3-(pyridin-4-yl)-6,7-dihydropyrazolo[1,5-*a*]pyrazin-4(5*H*)-one scaffold (Figure 13). Binding studies with the corresponding non-radiolabeled analogues in Glu2-HEK293 and Glu3-HEK293 cells yielded pIC_50_ values of 8.1–8.8 and E_max_ values close to 100%. Both ex vivo biodistribution and PET scans showed that [^18^F]-**1** and [^18^F]-**2** achieved high brain uptake with low activity in the pons, a region lacking mGluR2/3 expression, consistent with receptor-specific binding. [^18^F]-**2** displayed rapid washout but suffered from extensive defluorination and bone uptake, whereas [^18^F]-**1** exhibited steadily increasing cortical and striatal activity, likely reflecting high affinity or pseudo-irreversible binding. In both cases, pretreatment with a reference NAM selective for mGluR2/3 reduced brain uptake to pons levels, confirming target specificity.

#### 2.2.2. Metabotropic Glutamate Receptor 2 (mGluR2)

Research efforts have progressively shifted toward selective targeting of mGluR2 over mGluR3 to improve therapeutic outcomes. The development of PET radioligands targeting mGluR2 has evolved from early group II orthosteric antagonists, which showed several limitations—including poor BBB penetration, off-target binding, and interaction with efflux transporters—and were therefore mainly restricted to in vitro autoradiography or rodent imaging studies. These drawbacks prompted the shift toward AMs as a more promising strategy. Notably, mGluR2 PAMs have been explored in the context of pain, schizophrenia, and drug addiction, while NAMs have been associated with cognitive disorders such as AD. Despite their potential, none of the mGluR2 PET tracers has yet reached clinical use. The only structurally disclosed tracer tested in humans, [^11^C]JNJ-42491293, showed unexpected myocardial accumulation and off-target brain binding [112].

**Figure 14:**
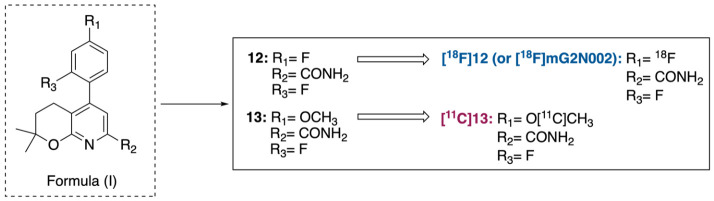
Chromane-based PET radioligands for mGluR2 imaging, with representative examples [^18^F]**12** or [^18^F]mG2N002 (blue) and [^11^C]**13** (purple) [113].

Following this line of research, a January 2023 published patent describes a new class of allosteric PET tracers based on the bicyclic structural scaffold of 3,4-dihydro-2*H*-pyrano|2,3-*b*]pyridine [113]. Two reference compounds emerged as suitable PET imaging candidates: 5-(2,4-difluorophenyl)-2,2-dimethyl-3,4-dihydro-2*H*-pyrano[2,3-*b*]pyridine-7-carboxamide (**12**, Figure 14) and 5-(2-fluoro-4-methoxyphenyl)-2,2-dimethyl-3,4-dihydro-2*H*-pyrano[2,3-*b*]pyridine-7-carboxamide (**13**, Figure 14). Both derivatives acted as potent mGluR2 NAMs (**12**: IC_50_ = 6 nM and **13**: IC_50_ = 93.2 nM). Docking studies performed on an mGluR2 homology model confirmed the nanomolar affinity range, with stable allosteric poses stabilized by multiple hydrogen bonds and π–π interactions. Besides their mGluR2 binding, compounds **12** and **13** were also characterized for lipophilicity, plasma and microsomal stability, and interaction with P-glycoprotein (P-gp). Their physiochemical properties fell within the range associated with brain permeability, and the lack of P-gp interaction suggested favorable CNS pharmacokinetics. Prompted by these encouraging pharmacological and physicochemical profiles, compounds **12** and **13** were successfully radiolabeled, yielding the PET tracers [^18^F]**12** ([^18^F]mG2N002) and [^11^C]**13**, respectively (Figure 14) [113].

The first PET imaging study was carried out in Sprague Dawley rats, where [^11^C]**13** showed excellent brain permeability, with marked accumulation in mGluR2-rich regions such as the striatum, thalamus, cortex, hypothalamus, hippocampus, and cerebellum. The tracer displayed favorable kinetics, with most radioactivity cleared within 30 min, and a moderate-to-high level of specific binding, supporting its suitability as an mGluR2 PET probe. The second study was conducted in an AD mouse model, where [^18^F]mG2N002 demonstrated excellent brain permeability and a heterogeneous yet consistent distribution across brain regions enriched in mGluR2. To further validate the potential of [^11^C]**13** as an imaging tool, a third study was conducted in non-human primates (cynomolgus monkeys), representing a pivotal translational approach for investigating the etiology of human neuropsychiatric disorders such as schizophrenia and drug addiction. [^11^C]**13** confirmed its ability to generate high-contrast images for mapping mGluR2 distribution in the primate brain. Kinetic analysis confirmed selective tracer accumulation in mGluR2-rich brain regions, which was markedly reduced after pretreatment with the selective NAM VU6001966, thereby validating target-specific binding. Consistent findings across rodent and primate studies provide strong evidence that [^11^C]**13**, together with [^18^F]mG2N002, are credible PET ligands for imaging mGluR2.

**Figure 15:**
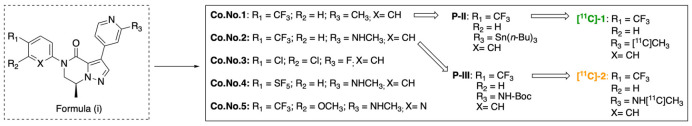
General scaffold of dihydropyrazolo[1,5-*a*]pyrazine derivatives from Janssen’s mGluR2 tracer series, with representative examples [^11^C]-**1** (green) and [^11^C]-**2** (orange) [114].

In 2021, Janssen Pharmaceutica NV revisited the class of 5-phenyl-3-(pyridin-4-yl)-6,7-dihydropyrazolo[1,5-*a*]pyrazin-4(5*H*)-ones previously disclosed in an earlier patent [111], introducing structural modifications to develop novel radiolabeled ligands selective for mGluR2 [114]. This invention covers a series of five compounds (Co. No. **1**–**5**, Figure 15) along with their corresponding precursors (P, Figure 15), allowing conversion into radiotracers labeled with either ^11^C or ^18^F. Compounds **1–5** demonstrated potent NAM activity at human mGluR2 in the [^35^S]GTPγS binding assay performed in the presence of glutamate. They showed pIC_50_ values in the range of 8.0–8.5 with E_max_ around 105–111%, and exhibited excellent selectivity over most mGluR subtypes, while discrimination against mGluR3 was more limited. Subsequent in vivo biodistribution studies in rats demonstrated that [^11^C]-**1** and [^11^C]-**2**, derived from the precursors P-II and P-III (corresponding to Co. No. **1** and Co. No. **2**, respectively; Figure 15), reached relatively high initial brain uptake at 2 min post-injection, followed by rapid clearance between 2 and 30 min. Although [^11^C]-**2** showed lower initial uptake than [^11^C]-**1**, its washout was slightly slower, suggesting improved retention. In vitro autoradiography further confirmed high mGluR2 specificity, with [^11^C]-**2** emerging as the most promising candidate by displaying the highest specific-to-nonspecific binding ratios and strong blockade by reference NAMs. While no PET imaging data have yet been reported, the favorable preclinical profile of [^11^C]-**2** highlights its potential as a promising candidate for future in vivo evaluation.

**Figure 16:**
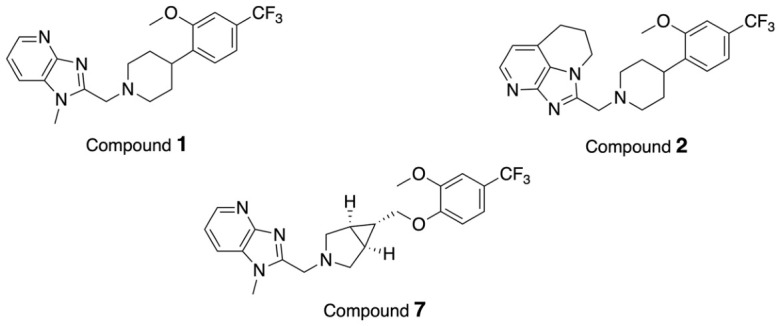
Representative pyridinimidazole-based compounds as mGluR2 PAMs and PET tracer candidates [115].

In the same year, another patent including a series of mGluR2 PAMs as PET ligands for investigating mGluR2-related pathophysiology at the molecular level was deposited by The General Hospital Corporation [115]. The application claims a broad series of benzimidazole-derived and related heterocyclic compounds, with radiolabeling achieved via one-step *O*-methylation of phenolic precursors using [^11^C]CH_3_I. Particular emphasis was given to compounds **1**, **2**, and **7** (Figure 16), which were profiled for molecular binding modes, pharmacology, physicochemical properties, and BBB permeability prior to in vivo evaluation, in order to mitigate the low and nonspecific brain uptake commonly observed with mGluR2 PET tracers. While all three derivatives showed solid profiles, compound **1** emerged as the most promising, combining nanomolar binding potency toward mGluR2 with excellent selectivity over other mGluRs, suitable lipophilicity and plasma protein binding, adequate metabolic stability, favorable passive permeability (PAMPA), and no P-gp liability. On this basis, compound **1** was selected for radiolabeling and subsequent in vivo evaluation as an mGluR2 PET radioligand. Ex vivo whole-body biodistribution of the [^11^C]-*O*-methylated analogue of compound **1** ([^11^C]**1**) showed rapid BBB penetration, combined hepatobiliary and renal clearance, and an overall pharmacokinetic profile consistent with use as a brain PET radioligand. In vivo PET studies in male Sprague-Dawley rats demonstrated time-dependent accumulation in mGluR2-rich regions, with the highest uptake in the thalamus, followed by striatum, cerebellum, and cortex. Specificity studies using a selective mGluR2 PAM from the same series confirmed target-specific binding, in line with the cAMP data on cold compound **1**. Moreover, self-blocking with compound **1** increased tracer accumulation by up to 49%, a potentiating effect that may open new therapeutic perspectives for mGluR2-related psychiatric and neurological disorders.

#### 2.2.3. Metabotropic Glutamate Receptor 4 (mGluR4)

To further broaden the scope of metabotropic glutamate receptor imaging, in 2022, the General Hospital Corporation disclosed novel mGluR4 PAMs, proposed as allosteric PET imaging probes [116]. mGluR4 is broadly distributed at synapses within the basal ganglia, predominantly at presynaptic sites, and is also found in the striatum, hippocampus, thalamus, and cerebellum. Its activation suppresses GABA and glutamate release in basal ganglia circuits and reduces excitatory transmission in the cortex, thus drawing attention to it as a possible therapeutic target in PD. Consistently, several mGluR4 PAMs, including foliglurax (tested in phase II clinical trials), have shown antiparkinsonian effects in preclinical PD models. In parallel, although multiple mGluR4 PET tracers have been developed, most of them faced significant limitations, including chemical and metabolic instability, rapid clearance and short half-life, as well as suboptimal imaging contrast.

**Figure 17:**
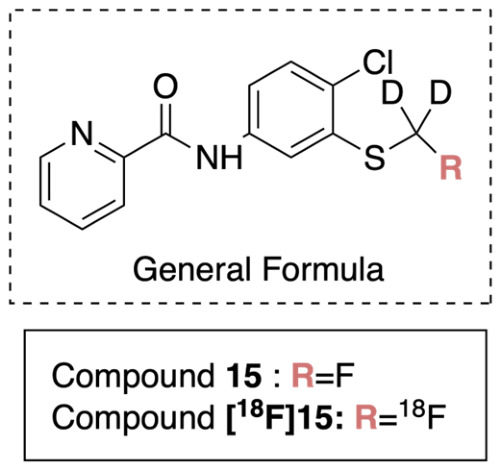
Chemical structure of [^18^F]**15** as an exemplary PET radioligand for mGluR4 [116].

The present invention discloses a series of picolinamide derivatives. Among them, compound **15** (Figure 17) stood out for its high mGluR4 affinity (IC_50_ = 3.4 nM) and PAM activity (EC_50_ = 324 nM), evaluated in the presence of an EC_20_ concentration of the reference agonist L-serine-*O*-phosphate (L-SOP) by monitoring intracellular cAMP levels. Selectivity was further assessed across different mGluR subtypes: mGluR1 and mGluR5 (G_q_-coupled) were tested using Ca^2+^ mobilization assays, while mGluR2, mGluR3, mGluR4, mGluR6, and mGluR8 (G_i/o_-coupled) were evaluated with cAMP assays. The results showed that compound **15** was selective over other mGluRs and displayed intrinsic mGluR4 agonist activity (EC_50_ = 2.75 μM), thereby classifying it as an mGluR4 ago-PAM, i.e., an allosteric modulator that exhibits intrinsic agonistic activity while enhancing the response to the endogenous ligand.

The in vitro pharmacological studies also showed that compound **15** possesses several CNS drug-like properties, including suitable lipophilicity and plasma protein binding, together with adequate metabolic and solution stability. Radiolabeling of compound **15** with fluorine-18 afforded [^18^F]**15** (Figure 17) in 10% radiochemical yield and 99% radiochemical purity, with a molar activity of 84.1 ± 11.8 GBq/μmol, likely influenced by the extended synthesis time. Ex vivo biodistribution studies demonstrated that the [^18^F]**15** displayed reversible binding across multiple tissues, including brain, liver, heart, lungs, and kidneys. In vivo PET imaging in male Sprague–Dawley rats confirmed brain accumulation in regions enriched with mGluR4. Pre-treatment with the corresponding non-labeled analogue **15** or with other mGluR4 allosteric ligands produced dose-dependent reductions in tracer uptake, confirming specific receptor binding. Collectively, these findings support the potential of [^18^F]**15** as a PET imaging probe for disorders involving mGluR4 dysfunction, such as PD.

#### 2.2.4. Dopamine D1 Receptor (D1R)

Dopaminergic neurotransmission plays a key role in the human brain being involved in movement, cognition and reward behavior. Five dopamine receptor subtypes (D1R―D5R) are known, and they have been classified into two major GPCR families. D1-like receptors (D1R and D5R) are primarily postsynaptic and preferentially couple to G_s_ proteins, leading to increased cAMP production, whereas D2-like receptors (D2R, D3R, and D4R) are expressed both pre- and postsynaptically and mainly signal through G_i/o_ proteins, resulting in reduced cAMP levels [117,118]. They are widely expressed across multiple brain regions: D1-like receptors are mostly found in the caudate–putamen (*striatum*), nucleus accumbens, *substantia nigra pars reticulata*, olfactory bulb, amygdala, and frontal cortex [119,120]. Accordingly, D1-like receptors are implicated in a variety of disorders, including negative symptoms in schizophrenia, attention-deficit/hyperactivity disorder (ADHD), PD and related movement disorders, dementia and AD, age-related cognitive decline, sleep disorders, and apathy. D2-like receptors are mainly expressed in the *striatum*, the lateral part of the *globus pallidus*, the core of the nucleus accumbens, the ventral tegmental area, the hypothalamus, the amygdala, the cortical areas, the hippocampus, and the pituitary [119,121]. While also involved in motor regulation (e.g., Huntington’s Disease (HD) and dystonia), D2-like receptors are more closely linked to schizophrenia (positive symptoms), reward-related disorders such as addiction, and endocrine dysregulation, particularly hyperprolactinemia.

**Figure 18:**
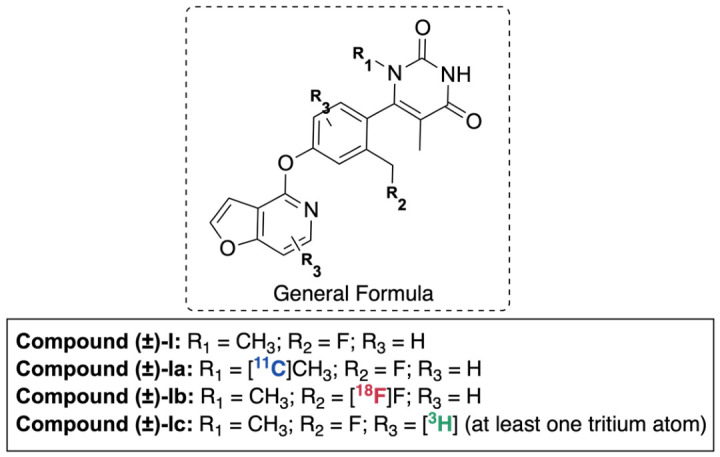
Structure of compound (±)-I, shown as a mixture of two atropisomers and alternatively radiolabeled with ^11^C ((±)-Ia), ^18^F ((±)-Ib), or ^3^H ((±)-Ic), with the corresponding labeling positions highlighted in blue, red, and green, respectively, disclosed by UCB Biopharma S.R.L. as D1-like PET imaging agents [123].

From a pharmacological perspective, the high degree of homology within the ligand-binding site among D1- and D2-like receptor subtypes poses a major challenge, as it often results in limited selectivity and off-target effects of existing drugs. This issue is further amplified in the development of PET imaging agents, where achieving high subtype selectivity is of critical relevance [122]. PET imaging agents developed so far for dopaminergic receptor subtypes are mainly based on agonists or, alternatively, antagonists; however, they are affected by several limitations, including insufficient subtype selectivity, suboptimal pharmacokinetic properties, and the generation of radioactive metabolites. The recent patent activity of UCB Biopharma S.R.L. has aimed to overcome these limitations by disclosing radiolabeled 6-[2-(fluoromethyl)-4-(furo[3,2-c]pyridin-4-yloxy)phenyl]-1,5-dimethylpyrimidine-2,4(1*H*,3*H*)-dione compounds, which act as selective D1-like PET imaging agents [123]. In detail, the present invention relates to compounds of formula (±)-I, existing as a mixture of two atropisomers (Figure 18), wherein at least one hydrogen atom is replaced by tritium (^3^H), the *N*-methyl group is labeled with ^11^C, or the fluorine atom is ^18^F. Compounds of formula (±)-I act as selective ligands for D1-like receptors, displaying high affinity for the human D5R (p*K*_i_ = 9.4) and exhibiting more than 1000-fold selectivity over the other 79 targets tested. Preclinical studies described in the patent show that the proposed D1-like PET tracers exhibit high and displaceable striatal binding in non-human primate brain, consistent with D1R distribution. In vivo PET imaging demonstrated favorable brain kinetics, quantifiable specific binding in the caudate and putamen, and a metabolite profile dominated by hydrophilic species unlikely to cross the BBB. Importantly, tracer uptake was markedly enhanced by co-administration of a D1R PAM, highlighting the suitability of these ligands for imaging D1R function. Although the compounds shown in Figure 18 act at the orthosteric site, these findings support the broader concept that allosteric modulation can provide an additional layer of selectivity for dopaminergic PET imaging, potentially overcoming limitations associated with the highly conserved orthosteric binding site.

### 2.3. Inflammatory Diseases

Inflammation is involved in many diseases, from immune-mediated disorders like rheumatoid arthritis (RA) to more common conditions such as cardiovascular disease and diabetes, beyond its close association with multiple aspects of tumor progression. As such, inflammation poses a significant diagnostic challenge, characterized by poorly defined clinical endpoints, high inter-patient variability, systemic involvement, and non-specific clinical or laboratory features. An example of this complexity is the potential mismatch between disease manifestation and treatment distribution, as observed in a pathology like RA that affects multiple tissues [124].

Consequently, the role of nuclear medicine in both the diagnostic workup and the therapeutic follow-up of these conditions has expanded considerably in recent years. Currently, the mainstay of inflammation imaging is [^18^F]FDG-PET/CT, routinely applied to a broad range of disorders, such as large-vessel vasculitis, sarcoidosis, and spondylodiscitis. However, its intrinsic limitation lies in the lack of specificity, since increased glycolytic uptake is also observed in malignant lesions and in *sterile* inflammatory processes [125,126].

Beyond immunoPET—which combines the high specificity and selectivity of monoclonal antibodies and engineered derivatives with the sensitivity and quantitative capabilities of PET imaging and is therefore increasingly at the forefront of clinical practice for several conditions, including inflammatory diseases—there is growing consensus that host-targeted approaches, such as GPCR-targeted tracers, may represent a relevant step forward to improve specificity in molecular imaging of inflammation [127,128]. After all, it is well known that GPCRs play key roles in inflammation, as they translate inflammatory states into molecular signals [129]. This makes them highly informative biomarkers for molecular imaging, offering the opportunity to decipher functional inflammatory pathways rather than merely detecting inflammatory burden. Consistent with this, tracers targeting chemokine-axis GPCRs are of particular interest.

#### 2.3.1. CC Chemokine Receptor 2 (CCR2)

CC-chemokine receptor 2 (CCR2) is a GPCR of the chemokine receptor family, primarily expressed on monocytes, immature dendritic cells and specific T-cell subsets, where it mediates chemotactic migration to sites of inflammation in response to endogenous ligands such as CCL2 [130]. The CCL2/CCR2 axis is essential for the recruitment of pro-inflammatory monocytes from hematopoietic sites to inflamed tissues; upon ligand stimulation, CCR2^+^ monocytes adhere to the endothelium and give rise to macrophages or dendritic cells, thereby amplifying local inflammatory responses through the release of pro-inflammatory mediators. Dysregulated or sustained activation of this pathway is implicated in a wide range of diseases, including inflammatory pain, atherosclerosis, neuroinflammatory disorders, RA and diabetic nephropathy.

It is worth noting that elevated levels of CCL2 and infiltration of CCR2^+^ immune cells are characteristic of acute and chronic lung diseases, including acute respiratory distress syndrome (ARDS), chronic obstructive pulmonary disease (COPD), experimental asthma, and pulmonary fibrosis. Together, these features make CCR2 a highly attractive molecular target for inflammation imaging, supporting the growing interest in the development of dedicated CCR2 imaging probes for both pre-clinical and clinical applications.

**Figure 19:**
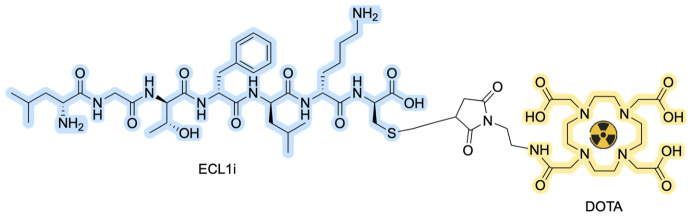
Chemical structure of ^64^Cu-DOTA-ECL1i [132,133].

In a June 2024 patent, inspired by the previously reported CCR2-binding peptide ECL1i (D-Leu-Gly-D-Thr-D-Phe-D-Leu-D-Lys-D-Cys) [131], later developed by Heo et al. in 2021 as the PET imaging probe ^64^Cu-DOTA-ECL1i (Figure 19) for imaging CCR2+ monocytes and macrophages in a heart transplantation mouse model [132], the applicants further investigated this monovalent tracer alongside a multivalent nanostructured probe consisting of ECL1i conjugated to copper-doped gold nanoclusters (^64^CuAuNCs-ECL1i) [133]. The authors demonstrated that peptide ECL1i enables specific in vivo PET/CT imaging of CCR2 in ischemia–reperfusion injury following lung transplantation, both as a monovalent peptide tracer and a multivalent nanoplatform. However, owing to their distinct pharmacokinetic profiles, ^64^Cu-DOTA-ECL1i is reported to be more suitable for rapid and serial CCR2 imaging, whereas the multivalent ^64^CuAuNCs-ECL1i, characterized by extended pharmacokinetics, is favored for long-term CCR2 detection and potential targeted theranostic applications.

To explore the ability to image CCR2-associated inflammation, ^64^Cu-DOTA-ECL1i was tested in LPS-induced lung injury, where its uptake increased during the acute phase only, with no signal detected in CCR2-deficient mice and complete blockade upon co-administration of non-radioactive ECL1i.

Beyond data including tests of ^64^Cu-DOTA-ECL1i specificity, sensitivity, and safety in mouse models of lung injury to validate radiotracer performance, the patent also reports ex vivo validation of CCR2 targeting in human tissues. Autoradiography on lung sections demonstrated markedly increased ^64^Cu-DOTA-ECL1i binding in samples from patients with COPD compared with normal donor tissues, consistent with increased CCR2 expression.

The patent further reports preclinical toxicology and dosimetry studies in animals, alongside the establishment of chemistry, manufacturing, and controls (CMC) documentation and standard operating procedures for ^64^Cu-DOTA-ECL1i. These activities are described as preparatory steps toward an exploratory IND application, thereby supporting the translational potential of the tracer.

#### 2.3.2. CXC Chemokine Receptor 2 (CXCR2)

The human CXC chemokine receptor 2 (CXCR2) is a GPCR involved in neutrophil-driven inflammatory responses. Neutrophils are key effectors of innate immunity, playing a central role in phagocytic host defense. Accordingly, variations in neutrophil recruitment and activation represent important biomarkers that can be quantified and correlated with disease presence and progression [134,135].

**Figure 20:**
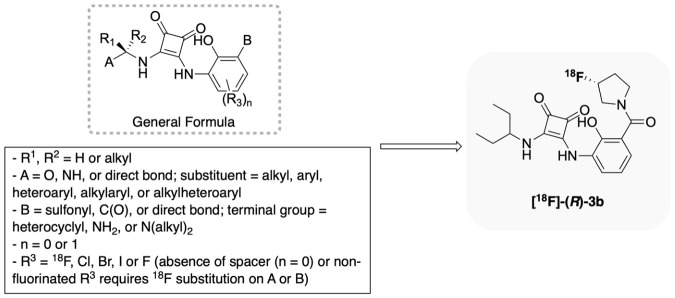
Structure of the first ^18^F-labeled CXCR2 PET tracer (**right**) together with the general formula (**left**) of its chemical class [136].

Researchers at the University of Würzburg have reported the first PET radiotracer targeting CXCR2, outlining its potential in vivo application for imaging CXCR2-mediated pathologies. These may include inflammatory conditions characterized by neutrophil recruitment, such as cancer, ischemic injury, bowel disease, respiratory disorders, atherosclerosis, and selected autoimmune or degenerative diseases [136]. The patent describes the development of fluorinated analogues based on a squaramide scaffold (general formula shown in Figure 20), obtained through exploration of different substitution sites and synthetic strategies. SAR analysis highlighted the critical role of hydrogen-bond donors in receptor recognition, with the aliphatic side chain of one donor being essential for maintaining CXCR2 selectivity, while a broad range of aliphatic and aromatic substitutions on the right-hand side were tolerated without significant loss of affinity. The resulting compounds were evaluated in vitro, leading to the identification of compound **3b** as the most suitable tracer candidate. In parallel, only a modest enantioselective preference was observed, as the (*R*)-enantiomer of **3b** (IC_50_ = 273 nM) slightly outperformed the corresponding (*S*)-enantiomer (IC_50_ = 428 nM). Thus, the ^18^F-labeled analogue of (*R*)-**3b** was selected for further development. Cell uptake studies demonstrated that [^18^F]-(*R*)-**3b** (Figure 20) exhibited time-dependent and CXCR2-specific accumulation in CXCR2-overexpressing HEK293 cells, which was effectively blocked by a reference CXCR2 antagonist. Tracer uptake was observed exclusively in CXCR2-positive cells, confirming high target specificity and highlighting the potential of [^18^F]-(*R*)-**3b** for functional imaging of neutrophils in inflammatory diseases. These findings were further corroborated in human peripheral blood neutrophils, where target specificity was again confirmed by antagonist blockade.

Initial microPET studies showed rapid clearance from most organs, short liver retention, and predominant renal excretion. Notably, negligible bone uptake—commonly associated with in vivo defluorination—indicated high metabolic stability. Collectively, these data provided key information for dosimetry estimation and the establishment of clinical imaging protocols.

#### 2.3.3. CXC Chemokine Receptor Type 4 (CXCR4)

CXCR4, previously discussed as a key molecular imaging target in oncology, has also emerged as a highly relevant target in inflammatory diseases due to its pivotal role in chemotaxis and leukocyte trafficking. In RA, the SDF-1/CXCR4 signaling axis mediates the pro-inflammatory migration of activated T cells to sites of inflammation; accordingly, synovial tissues from RA patients show increased infiltration of T cells with elevated CXCR4 expression [137]. Beyond autoimmune disorders, dysregulation of the SDF-1/CXCR4 axis also contributes to cardiovascular inflammatory pathologies. In the early stages of atherosclerosis, SDF-1/CXCR4 signaling promotes the recruitment of endothelial progenitor cells to sites of vascular injury, thereby participating in plaque formation, although some evidence suggests a potential atheroprotective role. Notably, atherosclerotic plaques are characterized by hypoxic microenvironments, which are known to upregulate CXCR4 expression and modulate immune cell trafficking. Accordingly, PET diagnostic agents targeting CXCR4 may provide valuable prognostic information in atherosclerosis [138]. On the whole, these observations highlight a significant unmet need for improved imaging agents and radiotherapeutic compositions enabling in vivo PET diagnosis and treatment not only of cancer but also of inflammatory/autoimmune diseases characterized by CXCR4 overexpression.

To the best of our knowledge, CXCR4-targeted imaging agents have been developed based on radiolabeled monoclonal antibodies, cyclam-derived inhibitors, and peptides. More recently, applicants from The University of British Columbia disclosed novel CXCR4-targeting radiopharmaceuticals derived from the cyclic peptide LY2510924 (cyclo[Phe-Tyr-Lys(iPr)-D-Arg-2-Nal-Gly-D-Glu]-Lys(iPr)-NH_2_), a potent CXCR4 antagonist capable of blocking SDF-1α binding with nanomolar affinity. These agents were extensively evaluated in PET studies using CXCR4-expressing malignancy models; however, the authors also claim their use for imaging inflammatory and autoimmune conditions [139,140].

#### 2.3.4. G Protein-Coupled Receptor 84 (GPR84)

**Figure 21:**
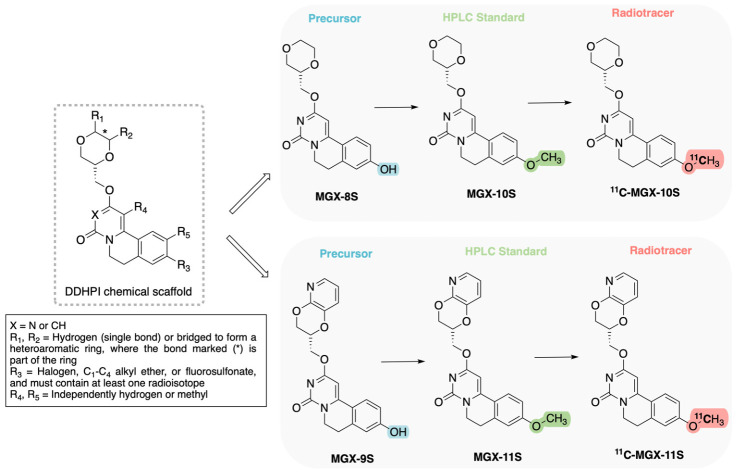
General formula of (1,4-dioxan-2-ylmethoxy)-6,7-dihydropyrimido[6,1-*a*]isoquinolin-4-one (DDHPI) derivatives patented as GPR84 radioligands. Representative radiotracers ^11^C-MGX-10S and ^11^C-MGX-11S together with their corresponding HPLC standards (MGX-10S, MGX-11S) and precursors (MGX-8S, MGX-9S) [142].

Among the GPCRs attracting increasing attention, the orphan receptor GPR84 has emerged as a promising PET imaging biomarker of detrimental innate immune activation. While its basal expression on pro-inflammatory myeloid cells such as macrophages, microglia, and neutrophils is low, it becomes strongly upregulated in response to CNS injury or inflammatory stimuli. To validate GPR84′s potential, a patent application filed in October 2024 by The Board of Trustees of the Leland Stanford Junior University disclosed the design and synthesis of ^11^C-labeled PET tracers for the selective imaging of neurological and inflammatory-related diseases associated with GPR84 expression [141]. The tracer selection was guided by prior research on (1,4-dioxan-2-ylmethoxy)-6,7-dihydropyrimido[6,1-*a*]isoquinolin-4-one (DDHPI) compounds (general formula in Figure 21), identified as potent and selective GPR84 NAMs [142]. Among the candidates, compounds with favorable physicochemical features for ^11^C-labeling and CNS penetration—such as molecular weight, topological polar surface area (TPSA), and lipophilicity—were prioritized. This led to the selection of MGX-10S and MGX-11S, which displayed low-nanomolar binding affinity in competitive assays with ^3^H-G9543, a reference GPR84 inhibitor. Functional cAMP and ^35^S-GTPγS assays confirmed target engagement, showing moderate-to-high nanomolar IC_50_ values, consistent with strong binding but limited inhibitory activity, a profile considered suitable for PET tracers. The non-labeled analogues (MGX-8S and MGX-9S) were synthesized as HPLC standards for radiotracer identification and subsequently radiolabeled to obtain ^11^C-MGX-10S and ^11^C-MGX-11S. In vitro assays in *h*GPR84-HEK293 cells demonstrated that ^11^C-MGX-10S displayed higher specificity than ^11^C-MGX-11S, with binding effectively blocked by the reference antagonist GLPG1205. In mice, both tracers crossed the BBB, but ^11^C-MGX-10S exhibited superior brain uptake and more favorable washout kinetics, prompting its further evaluation in a model of innate immune activation. In this setting, ^11^C-MGX-10S proved metabolically stable, remaining largely intact in the brain even under inflammatory conditions. PET imaging in LPS-treated mice revealed elevated ^11^C-MGX-10S uptake in the brain, liver, and intestines compared to controls, with increased binding across most brain regions, consistent with GPR84 upregulation in inflammation. Pre-treatment with the antagonist GLPG1205 significantly reduced tracer accumulation, confirming binding specificity, while ex vivo analyses supported the in vivo findings. Importantly, when compared with the TSPO tracer ^11^C-DPA-713, ^11^C-MGX-10S showed a superior signal-to-background ratio, underscoring its potential for sensitive detection of inflamed tissues.

Overall, this elegant patent has led to the development of ^11^C-MGX-10S as a highly specific GPR84 radiotracer, showcasing its strong in vivo stability, selective binding, and enhanced uptake in inflamed brain regions, positioning it as a valuable tool for neuroinflammation and innate immune activation imaging.

## 3. Discussion

The last five years have been characterized by an intense patent activity in the field of GPCR-targeted PET radiotracers. In some cases, the translational relevance of the reported compounds may be difficult to fully assess, particularly when PET applications are proposed primarily based on in vitro affinity or preliminary pharmacological data without comprehensive in vivo validation, metabolite analysis, or longitudinal imaging studies. Nevertheless, these patents often represent valuable early-stage platforms for radiopharmaceutical optimization, highlighting the importance of distinguishing proof-of-concept systems from more extensively validated radiotracers. This distinction is particularly relevant because, unlike conventional drug development, where a molecular scaffold generally converges toward a more defined pharmacological identity, the same scaffold in radiopharmaceutical development may be adapted to different radionuclides, diagnostic and therapeutic applications, and pharmacokinetic profiles through linker or chelator modulation, while also serving as part of a theranostic pair. As such, a radiopharmaceutical can simultaneously function as a molecular targeting vector, a radiochemical entity, and even a modular diagnostic–therapeutic platform. Consequently, intellectual property strategies in this field often prioritize versatile molecular platforms prior to extensive biological and imaging validation. Such patenting strategies also reflect the intrinsically iterative nature of radiotracer development, in which favorable in vitro profiles undoubtedly constitute an important prerequisite, although progressive validation through pharmacokinetic assessment, metabolic stability studies, off-target evaluation, and in vivo imaging investigations across complementary preclinical models are generally required to establish translational applicability and experimental reproducibility.

A critical analysis of the reviewed patents reveals primarily a recurrent a posteriori design strategy, whereby established GPCR ligands are adapted to PET imaging rather than conceived as de novo radiotracers. In many cases, development starts from known drugs, tool compounds, or clinically validated vectors, which have been modified to enable radiolabeling and then evaluated for their suitability as imaging agents. This pattern is consistently observed across multiple targets. For instance, WO2021130329 uses R54 as a structural template for the development of CXCR4-targeted tracers [72]. Similarly, WO2022023539 relies on a clinically mature ligand (PP-F11N) to develop a specific PET imaging agent for CCKBR-positive tumors [61]. Along the same lines, WO2025008409 discloses novel radiometal-based derivatives inspired by the well-known NK1R antagonist aprepitant [79]. Collectively, these examples illustrate a pragmatic and translationally oriented approach, in which prior knowledge of target engagement and in vivo behavior is leveraged to reduce development risk. Accordingly, the central design objective is the meticulous preservation of target affinity and selectivity while imposing the physicochemical and biological constraints required for successful PET imaging, including metabolic stability, clearance profile, and—where relevant—CNS penetration. Clear examples of this (radio)medicinal chemistry-driven rationale can be found in the development of chromane-based PET radioligands for mGluR2 imaging, where candidate selection follows a structured workflow encompassing binding mode analysis and stringent filtering based on lipophilicity, metabolic stability, and P-gp liability prior to radiolabeling and in vivo validation [113]. Similarly, the development of GPR84 PET tracers relies on the prioritization of compounds meeting the physicochemical requirements for CNS penetration, including molecular weight, TPSA, and lipophilicity compatible with brain imaging [141].

A careful analysis of patented imaging agents reveals the recurrent adoption of specific structural motifs that appear to be key determinants of in vivo performance, translational robustness, and theranostic potential. Across different disease areas and GPCR subclasses, several convergent design principles emerge.

Peptide-based radiotracers continue to dominate oncology-oriented patents, such as those targeting SSTRs, CCK2R, CXCR4, and KISS1R. Only a few small-molecule ligands emerge as exceptions within this peptide-dominated landscape, particularly those targeting NK1R [79] and NTSR1 [96]. The widespread use of peptide-based radiotracers in peripheral oncologic imaging is attributed to favorable pharmacokinetics and specific tumor targeting characteristics [143]. Indeed, radiolabeled peptides occupy an intermediate space between small molecules and large biologics such as antibodies [144]. Similarly to small molecules, they are considered particularly advantageous imaging agents because they generally exhibit high tissue and tumor penetration, relatively low immunogenicity, and rapid clearance kinetics, thereby enabling high imaging contrast within minutes to hours after administration. In addition, they are often synthetically accessible and readily amenable to radiolabeling [145,146,147]. Like large biologics, radiolabeled peptides generally exhibit high receptor selectivity and tolerate structural modifications provided that the essential binding motif is preserved [148]. These advantages, together with the overexpression of peptide receptors on human cancer cells as molecular targets—particularly GPCRs, whose dysregulation is associated with numerous malignancies—have led the bulk of peptide radiolabeling for PET imaging to be oriented toward oncological applications [144,149]. Meanwhile, unlike lipophilic small-molecule radiotracers, radiolabeled peptides generally exhibit higher molecular weight and increased hydrophilicity. These distinct physiochemical properties restrict passive diffusion across cellular membranes and the BBB, thereby minimizing nonspecific background tissue accumulation while promoting rapid renal excretion [143,150,151,152,153]. In addition, their generally hydrophilic character may contribute to limiting extensive plasma protein binding, thus favoring target availability.

However, unmodified linear peptides have a dark side. Most peptides exhibit a very short biological half-life because of rapid proteolysis by circulating enzymes [150]. Consequently, considerable efforts have been devoted to the introduction of structural modifications aimed at improving in vivo stability and pharmacokinetic behavior, including conformational constraints (e.g., cyclization and disulfide bridges) as well as the incorporation of non-natural amino acids. For example, the insertion of D-amino acids, known to be less susceptible to enzymatic degradation, is widely employed to improve enzymatic stability [154]. This strategy is exemplified by SST peptide analogs; for instance, both the introduction of D-amino acids and the reduction in the ring size to the bioactive core sequence led to the development of octreotide, an eight-amino-acid SST analog that preserves the four-amino-acid receptor-binding motif (Phe-D-Trp-Lys-Thr) of native SST-14 while exhibiting a markedly longer plasma half-life compared with endogenous SST (approximately 1.5–2 h for octreotide vs. 2–3 min for native SST) [143,155]. Another representative example is provided by the kisspeptin-derived conjugate platform disclosed in patent [87], in which several non-natural and chemically modified amino acid residues, including 2-amino-2-indancarboxylic acid (Aic), biphenylalanine (Bip), *N*-methylated amino acids, aza-amino acids, and fluorinated phenylalanines, were introduced to modulate receptor affinity, metabolic stability, and pharmacokinetic properties. Also, the CCK2R-targeting gastrin analog PP-F11N emerged from extensive SAR studies demonstrating that the incorporation of peptide sequences containing more than five proteolytically more stable D-glutamic acid residues generated optimized analogs with higher tumor uptake, improved tumor-to-kidney uptake ratios, and greater serum stability compared with previously developed minigastrin analogs [156,157]. Curiously, PP-F11N was designed by replacing methionine with norleucine (Nle), a non-oxidizable isosteric amino acid that prevented oxidative degradation during production and storage and avoided the formation of lower-affinity oxidized analogs [60]. Among other things, PP-F11N subsequently underwent further structural optimization aimed at improving metabolic stability while preserving receptor recognition, notably through the incorporation of 1,2,3-triazole-based bioisosteric motifs as amide bond surrogates, as described in the related patents [62,63].

Another commonly exploited strategy is peptide cyclization, which can avoid degradation by limiting the formation of conformers susceptible to proteolytic enzymes as well as mask the *N*- or *C*-terminus of the peptide sequence, the primary cleavage sites of exoproteases [158]. Interestingly, cyclic peptides also adopt a more restricted conformational ensemble in solution, often enabling more efficient and selective binding to the active site of the desired target [159,160]. A similar approach was described for the cyclopeptidic CXCR4 ligands disclosed in patent [75], as well as in the SSTR3-targeting tracer described in patent [57]. The latter incorporates several complementary stabilization strategies, including the introduction of metabolically more stable D-amino acids (D-Phe and D-Trp) together with an NTAG moiety enabling the formation of a disulfide bridge, thereby conferring conformational rigidity and improved metabolic stability.

Beyond this local level of structural control, many of the patents examined point to a complementary design rational with a strong emphasis on modular architectures. This tendency is particularly evident in metal-based radiopharmaceuticals, which frequently adopt a conserved M*–C–L–V organization, where the radiometal (M*) is coordinated by a chelator (C) and connected to the targeting vector (V) through a dedicated linker (L). Within this framework, modularity arises from the functional decoupling of target engagement, pharmacokinetic behavior, and radiochemical properties, allowing different molecular components to be combinatorially adapted to fit the intended application. More classical examples of modular optimization within a conserved molecular scaffold are provided by the patents on PP-F11N [58,59,60,61], and the MC2R-targeting ligands disclosed in patent [81], where selective variation in the coordinated radiometal among ^68^Ga, ^177^Lu, or ^111^In, while maintaining the same targeting vector and chelator system, allows the same molecular platform to be adapted to imaging, biodistribution, or theranostic settings. Other patents further extend this more conservative form of modularity through broader combinatorial diversification strategies. This is well exemplified by patent [54], where extensive variation in chelators, radionuclides, linkers, and peptide-based targeting motifs enabled access to a remarkably broad spectrum of radiopharmaceutical conjugates. A similar strategy emerges in patent WO2024061483, where multiple chelator/linker combinations are explored around a minimal tetrapeptidic CCK2R-binding pharmacophore, further supporting the growing tendency toward adaptable radiopharmaceutical platforms designed to accommodate distinct imaging and theranostic purposes [64]. Interestingly, this modular strategy has also been applied to dual-targeting systems, as exemplified by patent [67], which describes tri-functional molecular architectures simultaneously incorporating SSTR2- and CCK2R-binding motifs.

Thus, one of the clearest consequences of this modular design philosophy resides in the possibility to tailor the same radiopharmaceutical platform to radionuclides with markedly different physical half-lives and decay properties according to the biological kinetics of the targeting vector. This trend is clearly reflected in the oncology-focused patents analyzed herein, where ^68^Ga dominates peptide-based imaging agents compared with more traditional PET radionuclides such as ^11^C and ^18^F [57,61,72,96,102]. Such predominance likely reflects the favorable interplay between the physical properties of ^68^Ga and the pharmacokinetic profile typically associated with peptide-based targeting vectors. Additional factors contributing to the widespread adoption of ^68^Ga include its simple coordination chemistry, enabling the formation of highly stable macrocyclic chelates, together with its accessibility via ^68^Ge/^68^Ga generators, independent of on-site cyclotron facilities [161,162]. Moreover, peptide-based targeting vectors also appear compatible with alternative PET radiometals such as ^64^Cu, whose longer physical half-life provides greater flexibility for imaging acquisition and pharmacokinetic studies, as exemplified by the SSTR2-targeting systems disclosed in patents [53,54]. This versatility further extends to theranostic applications through the well-established ^68^Ga/^177^Lu pairing, where ^68^Ga-based imaging supports patient selection and treatment planning prior to ^177^Lu-based radiotherapy. Consistently, several patents discussed herein further suggest that many peptide-based targeting vectors retain compatibility with therapeutic or dosimetric radiometals such as ^177^Lu, ^90^Y, ^225^Ac, and ^111^In while preserving clinically relevant biodistribution profiles [58,67,75,81,96].

In this modular context, chelator and linker identities emerge as critical determinants of in vivo performance. Chelator selection strongly influences the thermodynamic stability and kinetic inertness of the radiometal complex, while also enabling efficient radiolabeling under mild conditions, including low temperatures, low precursor concentrations, and near-physiological pH [7]. Different chelator classes have been strategically exploited, including macrocyclic systems such as DOTA and NOTA [67,72,81], and cross-bridged scaffolds such as sarcophagine [53]. Interestingly, the CXCR4-targeting systems disclosed in patent [72] highlighted how the chelator identity alone may substantially influence the tracer performance. Despite preserving both the same targeting motif and the linker, the NOTA-containing analogue displayed superior CXCR4 affinity, tumor uptake, clearance, and imaging contrast compared with the corresponding DOTA derivative.

On the other side, linkers interposed between the chelator and the targeting vector in metal-based radiopharmaceuticals must preserve receptor affinity while simultaneously enabling fine-tuning of physicochemical and pharmacokinetic properties. Commonly employed linkers include hydrocarbon chains, short amino acid sequences, and PEG spacers. While hydrocarbon-based linkers may increase the overall tracer lipophilicity, often resulting in slower hepatobiliary clearance and increased abdominal background signal, the latter two types of linkers are frequently exploited to achieve a more favorable balance between receptor-mediated uptake, renal clearance, and reduced nonspecific binding, ultimately improving target-to-background ratios [7]. As an example, the NTSR1-targeting radioligands disclosed in patent [96] demonstrated that the linker greatly impacts the tumor residence time of radionuclide and, more significantly, the wash-out kinetics of the radionuclide in normal organs such as blood, kidney, liver, and lung. In particular, the derivative containing a compact polyamine-based spacer showed the highest tumor uptake (AUC = 1885.4), whereas the PEG-containing analogue markedly reduced blood and kidney uptake (AUC = 12.7 and 47.7, respectively), ultimately improving tumor-to-background ratios [96]. More sophisticated approaches, including enzyme-responsive self-immolative linkers (e.g., cathepsin B-cleavable systems), designed to enable controlled payload release, have also emerged [52].

In contrast, the design of radiotracers for CNS imaging strongly favors drug-like molecular architectures [163]. Successful CNS PET tracer development consistently adheres to classical ADME-driven constraints—such as optimized lipophilicity, TPSA, and conformational rigidity—closely mirroring the criteria applied to CNS-active drugs. This concept is well reflected, for example, in the CNS-oriented GPR84 radiotracers developed within the context of inflammatory disorders, where the two analogues ^11^C-MGX-10S and ^11^C-MGX-11S both displayed physicochemical properties compatible with BBB penetration. Nevertheless, the slightly higher lipophilicity of ^11^C-MGX-11S was associated with increased nonspecific binding to parental HEK293 cell membranes, whereas ^11^C-MGX-10S retained a more selective binding profile, thus highlighting the narrow physicochemical window often required for successful CNS PET tracer development [141].

CNS-oriented radiotracers recurrently rely on well-established heteroaromatic and polycyclic scaffolds, including pyridinimidazoles, chromans, pyridinecarboxamides, and condensed bicyclic pyrazolo–pyrazine cores, whose conformational rigidity and tunable physicochemical properties are generally compatible with BBB penetration and CNS pharmacokinetic requirements [111,113,114,115]. Accordingly, the dominant design constraints in this setting revolve around minimizing structural perturbation upon radiolabeling, carefully controlling lipophilicity, and preserving BBB permeability. Consequently, radionuclide selection is largely anchored to traditional isotopes such as ^11^C and ^18^F, favoring straightforward covalent labeling strategies (e.g., ^18^F-C bond formation).

The CNS imaging patent landscape reflects a mature and increasingly sophisticated view of GPCR biology. Several patents emphasize allosteric or non-competitive binding modes [113,116] or dual-targeting strategies [67], reflecting a growing interest in interrogating GPCR functional and signaling states rather than receptor density alone. This trend clearly shows the intention to evolve the field of GPCR-targeted PET imaging towards increasingly functional and mechanism-oriented applications. Future radiotracers will likely be gradually called upon to consolidate their strategic role as companion tools in drug development, supporting target validation, dose selection, and go/no-go decisions.

In addition to oncological and CNS applications, PET imaging targeting GPCRs in inflammatory disorders is an attractive, yet still largely underexplored, area. Inflammation-related PET imaging biomarkers offer unique opportunities to capture dynamic cellular activation and microenvironmental changes in vivo and may represent a unifying imaging axis across oncology and CNS, given the central role of inflammatory processes—either immune-driven or arising from non-immune tissue stress—in both tumor progression and neuroinflammatory disorders. This key strength of inflammation imaging aligns particularly well with GPCRs, whose biology is intrinsically linked to inflammatory signaling and immune-related mechanisms, thereby enabling a truly cross-cutting biomarker role. Established examples include GPCRs used as inflammatory imaging biomarkers, such as chemokine receptors from the CC and CXC families, which also serve as biomarkers of tumor behavior. More recently identified GPCRs, such as GPR65, have further expanded this concept, with GPR65 being reported as a novel immune-related biomarker whose role in regulating tumor-associated inflammatory responses could confer both prognostic and diagnostic relevance in lung adenocarcinoma [164].

Therefore, the limited number of patent filings related to GPCR-targeted probes for inflammation imaging is not due to a lack of biological relevance, but rather to the intrinsic complexity of inflammatory processes that are finely tuned systems. Nevertheless, as our understanding of inflammation biology continues to evolve and imaging strategies increasingly shift toward functional readouts, GPCR-targeted molecular imaging is well-positioned to deliver mechanistic and clinically relevant insights across inflammatory, oncological, and CNS-related conditions.

## Data Availability

No new data were created or analyzed in this study.
